# Reverse water gas-shift reaction product driven dynamic activation of molybdenum nitride catalyst surface

**DOI:** 10.1038/s41467-024-47550-8

**Published:** 2024-04-10

**Authors:** Hui Xin, Rongtan Li, Le Lin, Rentao Mu, Mingrun Li, Dan Li, Qiang Fu, Xinhe Bao

**Affiliations:** 1 State Key Laboratory of Catalysis, Dalian Institute of Chemical Physics, iChEM, Chinese Academy of Sciences, Dalian, 116023 China; 2 Analytical & Testing Center, Sichuan University, Chengdu, Sichuan 610064 China; 3 Key Laboratory of Green Chemistry and Technology, Ministry of Education, College of Chemistry, Sichuan University, Chengdu, Sichuan 610064 China

**Keywords:** Heterogeneous catalysis, Catalytic mechanisms, Chemical engineering

## Abstract

In heterogeneous catalysis catalyst activation is often observed during the reaction process, which is mostly attributed to the induction by reactants. In this work we report that surface structure of molybdenum nitride (MoN_*x*_) catalyst exhibits a high dependency on the partial pressure or concentration of reaction products i.e., CO and H_2_O in reverse water gas-shift reaction (RWGS) (CO_2_:H_2_ = 1:3) but not reactants of CO_2_ and H_2_. Molybdenum oxide (MoO_*x*_) overlayers formed by oxidation with H_2_O are observed at reaction pressure below 10 mbar or with low partial pressure of CO/H_2_O products, while CO-induced surface carbonization happens at reaction pressure above 100 mbar and with high partial pressure of CO/H_2_O products. The reaction products induce restructuring of MoN_*x*_ surface into more active molybdenum carbide (MoC_*x*_) to increase the reaction rate and make for higher partial pressure CO, which in turn promote further surface carbonization of MoN_*x*_. We refer to this as the positive feedback between catalytic activity and catalyst activation in RWGS, which should be widely present in heterogeneous catalysis.

## Abstract (web summary)

Catalyst activation commonly occurs during reactions. This study demonstrates that the surface’s active structure in nitride catalysts during the reverse water gas-shift reaction varies with the partial pressure of reaction products, resulting in enhanced catalytic activity through positive feedback between catalytic activity and the evolution of MoNx’s active structure.

## Introduction

Designing advanced heterogeneous catalysts is of crucial importance for energy conversion and chemical production, which relies on controlling the surface active sites but still remains challenging^1–5^. It is generally recognized that each component of a reaction including reactant, product, and sometimes intermediates may interact with the catalyst, which can induce various structure changes^6–14^. Recently, competitive and/or synergistic effects of reaction components on catalyst structure have been observed, resulting in more complicated structure dynamics during reactions^15–21^. The restructuring of a catalyst usually leads to catalyst activation/deactivation^22–28^ and subsequent change of local gaseous microenvironment surrounding the catalyst. In other words, the partial pressure or concentration of reactants and products near catalyst surface can be continuously varied by several orders of magnitude along with the catalyst surface restructuring during reaction process, i.e., higher partial pressure of the products for activation but higher partial pressure of the reactants in case of deactivation. The fluctuating local gaseous microenvironment would drive the catalyst restructuring again, which means that both surface active structure and local gaseous microenvironment are changing during the ongoing reaction^15,16,29–33^. Therefore, a dynamic interplay between catalytic activity and catalyst structure may exist in any reactions, which is the key to understand the dynamics of surface active sites.

Here, we investigate dynamic response between catalytic activity and structural evolution of molybdenum nitride (MoN_*x*_), an alternative to noble metal catalyst^34,35^, in reverse water gas-shift (RWGS) reaction. To simulate the reaction microenvironment under the condition from very low to high conversion, the reaction pressure is controlled from 1 mbar to 10 bar to adjust the partial pressure of reaction components. The corresponding surface active structures at the above reaction pressures are investigated by using (quasi) in-situ X-ray photoelectron spectroscopy (XPS). We find that the reaction products of CO and H_2_O competitively dominate the surface structural evolution under different reaction pressures. H_2_O-induced surface oxidation is prevailing at reaction pressure below 10 mbar. The effect of H_2_O and CO reaches balanced at 100 mbar making the surface almost keep at the pristine MoN_*x*_ structure. When further increasing reaction pressure, CO-induced surface carbonization of MoN_*x*_ to molybdenum carbide (MoC_*x*_) is dominant at 1 bar and above. Sequentially, the surface carbonization of MoN_*x*_ driven by CO enhances the reaction rate by two times compared with the fresh MoN_*x*_. Once the reaction happens the formed reaction products around the catalyst surface drive the formation of active MoC_*x*_ layers to enhance the catalytic activity and generate more reaction products, which displays a positive feedback between catalytic activity and evolution of active structure of MoN_*x*_ catalyst during RWGS.

## Results and discussion

### Reaction pressure-dependent surface structure of MoN_x_ catalysts

Tetragonal β-Mo_2_N catalyst was synthesized by a temperature-programmed nitridation process under H_2_/N_2_ (volume ratio of 3:1) mixed gases, followed by passivating under 1%O_2_/Ar for 12 h at room temperature^36^. We then tested CO_2_ hydrogenation performance over the as-prepared β-Mo_2_N catalyst at 500 °C and 1 bar (Supplementary Fig. 1), which shows high RWGS activity with CO_2_ conversion of 43.1% and CO selectivity above 90.2%.

**Fig. 1:**
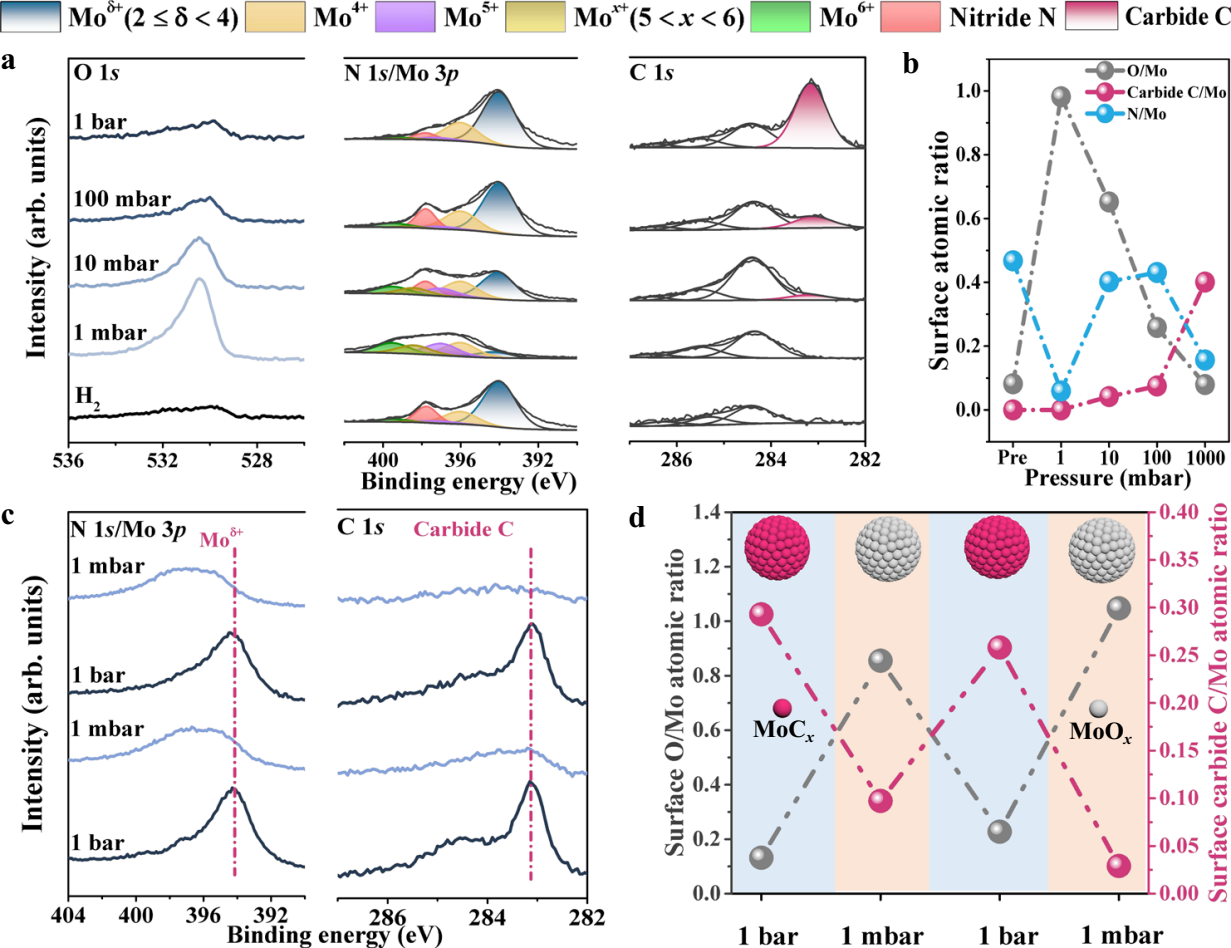
Surface structure of β-Mo_2_N catalyst in RWGS reaction with different pressures at 500 °C as measured by quasi in-situ XPS. **a** O 1*s*, N 1*s*/Mo 3*p*, and C 1*s* spectra acquired after reaction in 24%CO_2_/72%H_2_/N_2_ from 1 mbar to 1 bar. **b** Surface atomic ratios of O, carbide C, and N relative to Mo as function of reaction pressure. **c** N 1*s*/Mo 3*p* and C 1*s* spectra acquired after reaction in alternative 1 bar and 1 mbar 24%CO_2_/72%H_2_/N_2_ atmospheres (from bottom to top). **d** Surface O/Mo and carbide C/Mo atomic ratios calculated from C 1*s*, O 1*s*, and Mo 3*d* spectra in the cycled experiments with 1 mbar and 1 bar 24%CO_2_/72%H_2_/N_2_ atmosphere.

The surface structure in RWGS reaction has been investigated by (quasi) in-situ XPS in a near-ambient pressure XPS (NAP-XPS) system, which is composed of reaction and analysis chambers (Supplementary Fig. 2). Each catalyst is pre-reduced at 1 bar H_2_ and 500 °C to remove most of surface passivated oxide layers. XPS N 1*s*/Mo 3*p* spectra of the pre-reduced β-Mo_2_N catalyst (treated at 1 bar H_2_ and 500 °C) are displayed in Fig. 1a, which show the main peaks at 394.1 and 397.8 eV belonging to Mo^δ+^ (2 ≤ δ < 4) and N atoms in β**-**Mo_2_N structure^37^. In addition, a few small components at 396.0 ~ 399.5 eV are from molybdenum oxide species (MoO_*x*_, 2 ≤ x ≤ 3) which can hardly be removed even after the reduction treatment^37–39^ as confirmed by Mo 3*d* spectra in Supplementary Fig. 3 as well. A weak C 1*s* signal due to a small amount of residual contaminated carbon species also exists on the surface (Fig. 1a).

After exposing the pre-reduced catalyst to reaction gas at 1 mbar, the peaks belonging to Mo^δ+^ and N species in β-Mo_2_N almost disappear but those assigned to MoO_*x*_ species become dominant in N 1*s*/Mo 3*p* and Mo 3*d* spectra (Fig. 1a and Supplementary Fig. 3) along with a strong O 1*s* signal, indicating occurrence of the severe surface oxidation. However, upon increasing the pressure to 10 mbar, 100 mbar and 1 bar, the O 1*s* signal gets weaker accompanied with the weakened MoO_*x*_ peaks in N 1*s*/Mo 3*p* and Mo 3*d* spectra, which corresponds to less surface oxidation. Meanwhile, a new C 1*s* peak at 283.1 eV assigned to MoC_*x*_ appears and gradually gets strengthened^40^. The N 1*s* peak for nitride N species reappears at 10 mbar and gets stronger at 100 mbar, which almost disappears again at 1 bar.

The evolution of various surface Mo species has been further illustrated by plotting atomic ratios of carbide C (from MoC_*x*_), N (from MoN_*x*_), and O (from MoO_*x*_) relative to Mo as the function of reaction pressure (Fig. 1b). We observe the sudden increase in O/Mo but decrease in N/Mo with almost unchanged carbide C/Mo upon exposing the as-reduced β-Mo_2_N catalyst to 1 mbar reaction gas. The results indicate that surface N atoms coordinated with Mo atoms are substituted by O atoms under 1 mbar RWGS reaction gas. With increasing reaction pressure, the decreasing O/Mo but increasing carbide C/Mo have been observed while N/Mo starts to increase at 10 and 100 mbar and then decreases at 1 bar. As a result, MoC_*x*_ species dominate the surface under 1 bar reaction gas while the maximum surface MoN_*x*_ species exist under 100 mbar reaction gas. It can be speculated that the surface O atoms are removed and further replaced by C atoms, forming dominant surface MoN_*x*_ and MoC_*x*_ structures at 100 mbar and 1 bar, respectively. Mo atoms in MoC_*x*_ exhibit similar binding energy of Mo^δ+^ species in β-Mo_2_N^41^, and thus Mo 3*d* spectra are nearly unchanged after the surface carbonization.

The β-Mo_2_N catalyst has been subjected to the same treatment at 1 bar reaction gas but transferred in air for ex-situ XPS analysis. C 1*s* signal of carbides can be also observed but with much lower intensity after exposure to air for 5 min and totally disappears after exposure to air for 1 day (Supplementary Fig. 4), implying that the surface MoC_*x*_ species can form in the ambient pressure reaction which are sensitive to the oxidizing components (O_2_ and/or H_2_O) in air^24,42,43^ and form inert oxide overlayer on the surface. Besides, in-situ NAP-XPS experiments at 1 mbar have also been conducted, showing the similar results with quasi in-situ XPS results at 1 mbar, in which MoO_*x*_ species dominates the surface (Supplementary Fig. 5). These imply that the effect of reaction pressure can’t be ignored for the dynamic structural evolution.

We have shown that the surface structure of the nitride catalyst displays an interesting pressure dependence in the reaction gas. The surface has been further treated with 1 bar and 1 mbar reaction gases, alternatively. As shown in Fig. 1c and Supplementary Fig. 6, the surface dominated by MoC_*x*_ after exposure to 1 bar reaction gas gets oxidized at 1 mbar reaction gas and the surface MoO_*x*_ structure formed at 1 mbar is carbonized again at 1 bar reaction gas. Surface O/Mo and carbide C/Mo atomic ratios calculated from Mo 3*d*, O 1*s*, and C 1*s* (only the carbide species) peak areas reveal this reversible tendency more clearly (Fig. 1d). At 1 bar reaction gas, high carbide C/Mo but low O/Mo surface atomic ratios are observed, while this case gets reversed at 1 mbar pressure. Thus, the surface oxidation and carbonization processes can be well controlled by changing the reaction gas pressures between 1 mbar and 1 bar.

### Driving force for structural evolution of MoN_*x*_

**Fig. 2:**
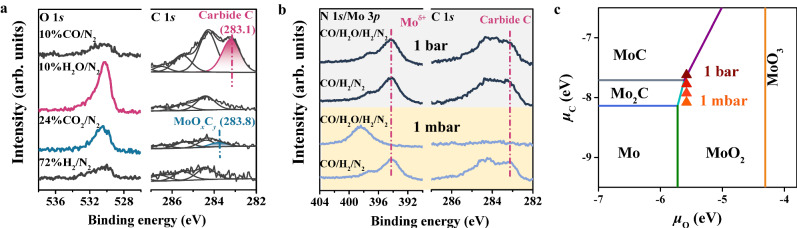
Effect of each reaction component on surface structural evolution of β-Mo_2_N. **a** Quasi in-situ XPS O 1*s* and C 1*s* spectra of the pre-reduced β-Mo_2_N exposed to 1 bar 72%H_2_/N_2_, 1 bar 24%CO_2_/N_2_, 1 bar 10%H_2_O/N_2_, and 1 bar 10%CO/N_2_, respectively, at 500 °C for 30 min. **b** Quasi in-situ XPS N 1*s*/Mo 3*p* and C 1*s* spectra measured under 1 mbar (yellow region) and 1 bar (grey region) CO/H_2_/N_2_ and CO/H_2_O/H_2_/N_2_ atmospheres, respectively. At each pressure, the pre-reduced β-Mo_2_N was treated in CO/H_2_/N_2_ and then exposed in CO/H_2_O/H_2_/N_2_. The ratio of H_2_ is 62%; CO and H_2_O in the mixed atmospheres are all 10%, similar to that in real reaction environment at 500 °C. **c** The computed phase diagram of Mo metal, carbides, and oxides. Each solid line denotes the boundary between two domains (phase equilibrium). The Mo species with the lowest free energy is marked for each domain. The triangles represent the position of *μ*_o_ and *μ*_c_ within specific reaction equilibrium conditions (details seen in Supplementary Table 1).

To understand the structural change of the nitride surface in the reaction, the pre-reduced β-Mo_2_N catalyst is exposed to each reaction component in RWGS i.e., 72%H_2_/N_2_, 24%CO_2_/N_2_, 10%H_2_O/N_2_, and 10%CO/N_2_ at 1 bar and 500 °C for 30 minutes (the amount of CO and H_2_O are calculated based on the CO_2_ conversion results at 500 °C) and then quasi in-situ XPS measurements have been carried out (Fig. 2a). In 72%H_2_/N_2_ gas the catalyst surface keeps unchanged. In case of exposure to 10%H_2_O/N_2_, there are oxides on the surface as indicated by the appearance of a strong O 1*s* peak and a positive binding energy shift of N 1*s*/Mo 3*p* signals (Supplementary Fig. 7). These manifest that product H_2_O is more likely to oxidize the β**-**Mo_2_N surface. When exposing the catalyst to 10%CO/N_2_, a strong peak at 283.1 eV from carbides appears in C 1 *s* spectra along with the weakened N signal in N 1*s*/Mo 3*p* spectra (Fig. 2a and Supplementary Fig. 7), indicating that CO can directly carbonize the β**-**Mo_2_N surface. In addition, the peak around 284.4 eV is also significantly enhanced, suggesting the surface accumulation of graphitic carbon atoms from CO via the Boudouard process^44^. This surface reaction is similar to CO-induced formation of metal carbides on Fe-based catalysts during RWGS reaction^16,45^. Notably, if this CO-treated sample is further exposed to 72%H_2_/N_2_ the carbide peak intensity has been increased, indicating that H_2_ can further promote the carbonization process (Supplementary Fig. 8). Upon exposing the β-Mo_2_N catalyst to 24%CO_2_/N_2_, a weak peak at 283.8 eV from oxycarbide (MoO_*x*_C_*y*_)^40,46^ appears in C 1*s* spectra and O 1*s* peak intensity has been slightly increased, implying that CO_2_ can slightly carbonize and oxidize the surface. Since nitride catalysts can catalyze dissociation of CO_2_ to CO (Supplementary Fig. 9)^47^, the dissociated CO and O from CO_2_ may cause the weak carbonization and oxidization, respectively.

The above results reveal that the reaction products H_2_O and CO govern the surface structure of β**-**Mo_2_N catalysts during reaction. To explore the effect of CO and H_2_O gases at different partial pressures, we have investigated the surface structure of the pre-reduced β-Mo_2_N catalyst exposed to CO/H_2_/N_2_ and CO/H_2_O/H_2_/N_2_ under both 1 mbar and 1 bar conditions at 500 °C ensuring that the partial pressures of CO, H_2_O and H_2_ are the same as that in the corresponding reaction conditions (Fig. 2b). At 1 mbar CO/H_2_/N_2_ the surface structure is dominated by MoC_*x*_ as confirmed by the obvious C 1*s* peak at 283.1 eV (Fig. 2b). From this surface, the exposure gas has been switched to 1 mbar CO/H_2_O/H_2_/N_2_. Interestingly, the C 1*s* peak at 283.1 eV disappears and the Mo 3*p* peaks shift to higher binding energy positions, suggesting the severe surface oxidation, which is also verified by the appearance of a strong O 1*s* signal (Supplementary Fig. 10). The result manifests that the oxidation by H_2_O surpasses the carbonization by CO at 1 mbar pressure. The surface carbides are also formed at 1 bar CO/H_2_/N_2_, which however remain unchanged with additional introduction of H_2_O into the CO/H_2_/N_2_ gas (Fig. 2b), demonstrating that the carbonization by CO becomes dominant at 1 bar pressure no matter the coexistence with H_2_O or not.

Apparently, both CO and H_2_O competitively interact with β**-**Mo_2_N, which are determined by varied oxygen and carbon chemical potentials at the different partial pressures. Figure 2c and Supplementary Fig. 11a display the phase diagram of bulk Mo-based compounds as a function of chemical potentials of oxygen (*μ*_o_) and carbon (*μ*_c_) obtained by density functional theory and ab initio thermodynamics calculations (see details in the Supplementary Information), which can be used to figure out the surface structure under the given conditions (triangles in the figure). Under all considered conditions H_2_O possesses a higher oxidation ability (more positive *μ*_o_) than CO_2_ (Supplementary Fig. 11b), which is consistent with the experiment results. When increasing the total reaction pressure but fixing ratios of all gas components, *μ*_o_ is unchanged but *μ*_c_ becomes more positive, which implies that *μ*_c_ may become more critical than *μ*_o_ in deciding the β**-**Mo_2_N structure. Under low reaction gas pressure surface oxygen species are difficult to be removed due to low partial pressure of CO (lower *μ*_c_ case), while high partial pressure CO can consume surface oxygen species and leave carbon species behind favoring the carbonization of β**-**Mo_2_N (higher *μ*_c_ case). Besides, the competition of CO and H_2_O would reach a balance for CO in moderate partial pressure (middle *μ*_c_ case) such that few carbon or oxygen species are left on β**-**Mo_2_N surface making β**-**Mo_2_N intact (~100 mbar in Fig. 1a). As for this, we also exclude the possibility of nitridation by N_2_ for the appearance of MoN_*x*_, as seen from unchanged C 1*s* and N 1*s*/Mo 3*p* signals when the spent β-Mo_2_N catalyst which treated at 1 bar reaction gas and 500 °C (denoted as β-Mo_2_N-1bar) is exposed to 1 bar N_2_ at 500 °C for 30 min (Supplementary Fig. 12). Overall, the thermodynamic scheme accompanied with experimental results show that increasing partial pressure of CO is in favor of carbonization of Mo_2_N surface, well explaining the surface oxidation of β-Mo_2_N below 10 mbar but the surface carbonization above 100 mbar.

### Dynamic activation of MoN_*x*_ catalyst by reaction product-induced carbonization

**Fig. 3:**
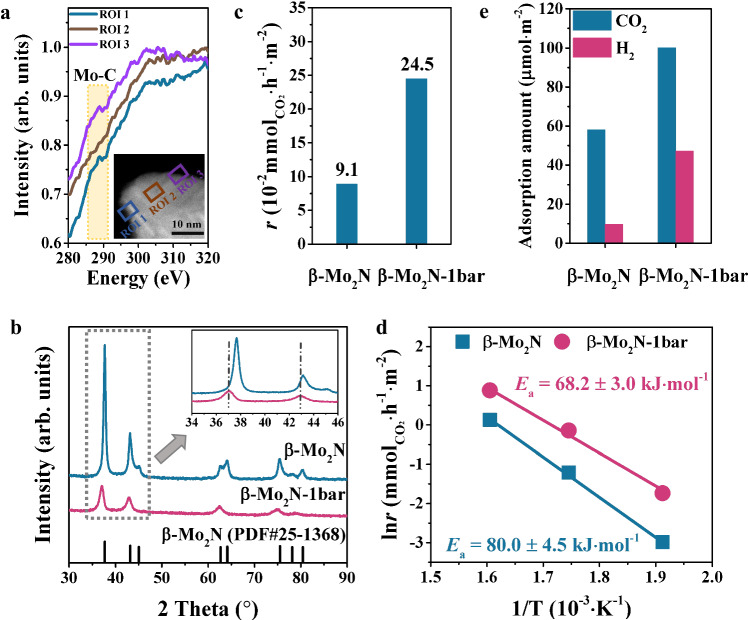
Structure identification of β-Mo_2_N catalyst after RWGS reaction at 1 bar and its corresponding catalytic activity. **a** EELS spectra over the β-Mo_2_N-1bar catalyst, which are recorded from regions indicated in the inset of HAADF-STEM image. **b** XRD patterns of the fresh β-Mo_2_N and β-Mo_2_N-1bar catalysts. **c** Reaction rate of CO_2_ conversion on the β-Mo_2_N catalysts normalized by the specific surface area under reaction condition of 50 mg catalysts, 250 °C, 24%CO_2_/72%H_2_/N_2_, and WHSV = 30,000 mL·g_catal_^−^^1^·h^−^^1^ with the CO_2_ conversion below 5%. **d** Arrhenius plots of the fresh and β-Mo_2_N-1bar catalysts with the CO_2_ conversion below 16%, which are measured from 250 to 350 °C. The test of kinetic results has been done below 350 °C since the surface carbonization starts to happen above 350 °C (Supplementary Fig. 13), which could show the activity difference of the MoN_*x*_ catalysts with and without surface carbonization. **e** The CO_2_ and H_2_ adsorption amount of fresh β-Mo_2_N and β-Mo_2_N-1bar catalysts calculated from CO_2_/H_2_-TPD experiments.

The oxidation of β**-**Mo_2_N catalyst in water gas-shift (CO + H_2_O) and RWGS reactions has been revealed to occur in the surface region, forming nitride@oxide nanostructures with enhanced activity^48–50^. Here, we have further revealed that β**-**Mo_2_N catalyst gets carbonized during RWGS reaction. In the following, structural characterization and catalytic performance test of the carbonized catalysts are performed. Scanning transmission electron microscopy coupled with electron energy loss spectroscopy (STEM-EELS) analysis of the β-Mo_2_N-1bar reveals carbon K edges from the edge regions (region of interest (ROI) 1, 3) and center region (ROI 2) of a β-Mo_2_N-1bar particle (Fig. 3a). At the edges, besides π* signal (about 285.4 eV) from graphite there is a peak at 288.6 eV ascribed to Mo-C σ* bond confirming the presence of MoC_*x*_^51,52^. However, this signal at 288.6 eV is missing in the center region. The EELS results demonstrate that the MoC_*x*_ species mainly form on the surface region.

X-ray diffraction (XRD) pattern of β-Mo_2_N-1bar catalyst shows that its diffraction peaks shift to lower angle compared with the fresh sample, which is consistent with the larger lattice fringe (2.46 Å) than the fresh β-Mo_2_N (2.38 Å) as observed in high-resolution TEM (HR-TEM) images (Fig. 3b and Supplementary Fig. 14). Thus, the structure of β-Mo_2_N-1bar catalyst is suggested to be MoC_*x*_ overlayers on β-Mo_2_N with few C atoms doped in the β-Mo_2_N bulk. Further increasing the reaction pressure to 10 bar, carbonization gets stronger and extends to the bulk as shown by the higher carbide C/Mo atomic ratio and the similar XRD pattern to α-MoC (Supplementary Fig. 15 and Fig. 16). This phenomenon is in line with the formation of MoC phase at higher carbon potential from the phase diagram (Fig. 2c).

Interestingly, the β-Mo_2_N−1bar catalyst shows higher RWGS activity (Supplementary Fig. 17). CO_2_ conversion rate of this catalyst is above 2 and 3 times those of the fresh β-Mo_2_N and α-MoC, i.e., 24.5 vs. 9.1 vs. 6.8 (10^−2^ ${{\mbox{mmol}}}_{{{\mbox{CO}}}_{2}}$·h^−1^·m^−2^) under the identical reaction conditions (Fig. 3c and Supplementary Fig. 18) when the catalytic activity is normalized by their specific surface area (Supplementary Table 2, 17.1, 21.5, and 108.7 m^2^/g for the β-Mo_2_N-1bar, fresh β-Mo_2_N, and α-MoC accordingly). In addition, the apparent activation energy (*E*_a_) for β-Mo_2_N-1bar is 68.2 ± 3.0 kJ·mol^−^^1^, which is lower than that of the fresh catalyst (80.0 ± 4.5 kJ·mol^−^^1^) (Fig. 3d). As CO_2_ and H_2_ show negligible effect on the surface structure of β-Mo_2_N (Fig. 2a), the CO_2_/H_2_-temperature programmed desorption (CO_2_/H_2_-TPD) experiments were conducted in order to illustrate surface adsorption and desorption of CO_2_ and H_2_. Figure 3e shows that the β-Mo_2_N-1bar catalyst has higher adsorption amount for both CO_2_ and H_2_ than fresh β-Mo_2_N. These manifest that the formed MoC_*x*_ overlayers on Mo_2_N surface improve the reactant adsorption, thus enhancing the catalytic activity. The CO-induced surface carbonization also occurs on a γ-Mo_2_N catalyst during the same CO_2_ hydrogenation reaction but with less enhancement of its catalytic activity compared with that of β-Mo_2_N catalyst (Supplementary Figs. 19, 20). This is due to the lower carbonization degree of γ-Mo_2_N in contrast with β-Mo_2_N because of the weaker chemical adsorption of CO on γ-Mo_2_N than that on β-Mo_2_N (Supplementary Figs. 21,  22 and Table 3).

### The interplay between catalytic conversion and catalyst structure

**Fig. 4:**
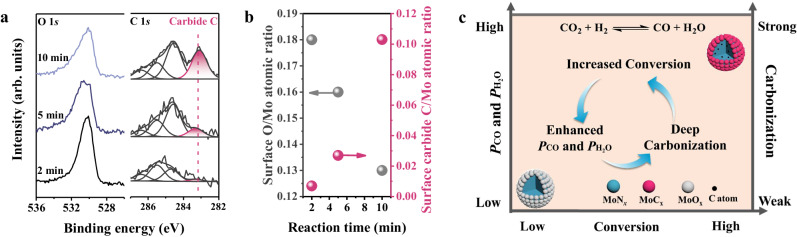
Structural evolution of β-Mo_2_N at 1 bar RWGS reaction atmosphere and 500 °C as measured by quasi in-situ XPS. **a** O 1*s* and C 1*s* spectra acquired after reaction for 2, 5, 10 min, accordingly. **b** Surface O/Mo and carbide C/Mo atomic ratios calculated from C 1*s*, O 1*s*, and Mo 3*d* spectra from Fig. 4a. **c** Schematic diagram of the positive feedback between catalytic activity and structural evolution of β-Mo_2_N under RWGS reaction.

To further reveal the structural evolution with the reaction activity, the surface structure of β-Mo_2_N has been investigated in the kinetic region of RWGS reaction at 1 bar and in a closed reaction cell, in which the reaction time represents degree of the reaction conversion. As shown in Fig. 4a, a strong O 1*s* peak already appears at reaction time of 2 min but carbide C 1*s* peak at 283.1 eV is almost invisible, indicating the dominant surface oxidation process at the initial stage of the reaction. With the reaction time increasing from 2 to 5 and 10 min, the O 1*s* peak intensity gets weakened and carbide C 1*s* peak is strongly strengthened. The decreased surface O/Mo and increased carbide C/Mo atomic ratios displayed in Fig. 4b further confirm the transformation from oxidized to carbonized surface with the ongoing reaction at 1 bar.

The results suggest that the surface structure of MoN_*x*_ dynamically responds to the local gaseous microenvironment with varying concentration of formed H_2_O and CO at different conversions. At the very beginning of the reaction, the low conversion with a small amount of H_2_O and CO products only induces the formation of surface MoO_*x*_ (2 min), which is similar to the case at reaction pressure below 10 mbar. After prolonging the reaction time, conversion would increase generating more products. The enriched H_2_O and CO products facilitate the formation of surface MoC_*x*_ (above 2 min), which is similar to the case at reaction pressure above 100 mbar. Therefore, it can be concluded that the reaction products ignite the surface restructuring (oxidation and carbonization) of MoN_*x*_, enhancing the RWGS activity and increasing the partial pressure of CO near MoN_*x*_ surface. The changing gaseous microenvironment in turn promotes the carbonization at the catalyst surface, which displays positive feedback between catalytic activity and structural evolution of MoN_*x*_ in RWGS reaction as illustrated by Fig. 4c. Beyond the famous pressure gap mostly considering one specific reaction gas or the reactant^19^, our findings demonstrate the importance of the response to products and catalytic activity in identifying the dynamics of active sites under catalytic conditions.

This work describes that the surface structure of MoN_*x*_ catalysts is mainly determined by reaction products i.e., CO and H_2_O in the RWGS reaction. When the reaction pressure is below 10 mbar the partial pressure of CO/H_2_O is naturally low, such that H_2_O dominates the formation of Mo oxide (MoO_*x*_) overlayer on MoN_*x*_. With the reaction pressure above 100 mbar, the increased partial pressure of CO enables the surface carbonization of MoN_*x*_, which suppresses the oxidization effect of H_2_O but produces Mo carbide (MoC_*x*_) overlayers from CO. The generated MoC_*x*_ overlayers enhances the reaction rate by two times compared with the pristine MoN_*x*_. Therefore, the emergence of reaction products drives the surface restructuring of MoN_*x*_ catalysts, enhancing activity and then increasing partial pressure of reaction products near MoN_*x*_ surface, which can further strengthen the surface carbonization of MoN_*x*_. Thus, positive feedback between catalytic activity and catalyst structure evolution during the reaction has been clearly demonstrated. This work highlights the dynamic interplay between local gaseous environment linked with catalytic activity and evolution of surface active structure during reactions.

## Methods

### Catalyst synthesis

Pure phase β-Mo_2_N catalyst was synthesized using reported methods in literatures^36,53^. First, MoO_3_ powder was obtained by calcination of ammonia heptamolybdate ((NH_4_)_6_Mo_7_O_24_·4H_2_O) at 500 °C for 4 h. After cooling down, the 0.5 g MoO_3_ powder was transferred into a quartz tube and then nitridized under the flow of H_2_ (100 mL/min)/N_2_ (45 mL/min) mixed atmosphere to obtain β-Mo_2_N. The detailed temperature programmed nitridation process was as follows: the temperature was linearly increased from ambient temperature to 300 °C with a heating rate of 5 °C·min^−1^, and then the heating rate was controlled at 1 °C·min^−1^ from 300 to 700 °C maintaining at 700 °C for 2 h. After cooling down to room temperature, the obtained Mo nitrides catalysts were passivated in 1%O_2_/Ar for 12 h to avoid violent oxidation upon exposure to air. The preparation method for γ-Mo_2_N is identical to that of β-Mo_2_N but using pure NH_3_ (100 mL/min) as the nitridation atmosphere^37,53^. The α-MoC was obtained via carbonization of γ-Mo_2_N under 20%CH_4_/H_2_ (100 mL/min) atmosphere^54^. The detailed temperature programmed carbonization process was as follows: the temperature was linearly increased from ambient temperature to 300 °C with a heating rate of 5 °C·min^−^^1^, and then the heating rate was controlled at 1 °C·min^−1^ from 300 to 700 °C maintaining at 700 °C for 2 h. After cooling down to room temperature, the obtained Mo carbide was passivated in 1% O_2_/Ar for 12 h to avoid violent oxidation upon exposure to air.

### Catalytic tests

CO_2_ hydrogenation reactions were tested using a homemade fixed-bed micro-reactor. The 50 mg pelleting catalysts (20 ~ 40 mesh) diluting with 200 mg SiC were loaded in a quartz tube with an inner diameter of 4 mm. The reactant gas consists of 24% CO_2_, 72% H_2_ (volume ratio), balanced with N_2_, which was used as an internal standard. Before each measurement, the catalysts were pretreated by 75%H_2_/N_2_ at 500 °C for 2 h to remove the impurities adsorbed on catalyst surface, and then switched to reaction gas with the weight hourly space velocity (WHSV) of 30,000 mL·g_catal_^−1^·h^−1^. The reaction started from 250 to 500 °C with interval of 50 °C at atmospheric pressure. Each temperature was maintained for 1 h. The effluent gas was online analyzed by Agilent GC6890N equipped with a TDX-1 column and thermal conductivity detector. The reaction kinetic parameters were obtained under low CO_2_ conversion (< 16%) by decreasing the reaction temperature.

### Catalyst characterization. X-ray diffraction (XRD)

The diffraction patterns of catalysts were performed using the Empyrean-100 diffractometer equipped with a Cu Kα radiation source (λ = 1.5418 Å) at 40 kV and 40 mA. The XRD patterns were collected with the 2θ value ranged from 30° to 90° at a scanning rate of 8°/min.

### Scanning Transmission Electron Microscopy (STEM)

HAADF-STEM image and electron energy-loss spectroscopy (EELS) elemental mapping were recorded on a JEM-ARM300F microscope operated at an accelerating voltage of 60 kV. The samples for TEM analysis were prepared by dropping ethanol suspension of samples on the carbon film-coated copper grids. High-resolution TEM (HRTEM) image was conducted on a JEM−2100 microscope operated at an accelerating voltage of 200 kV.

### Surface area of the catalysts

The textural properties of the samples were determined by N_2_ adsorption on a Quadrasorb evo sorption analyzer at liquid nitrogen temperature (−196 °C). Prior to the measurements, the catalysts were treated under vacuum at 300 °C for 4 h. The specific surface area was determined by the Brunauer-Emmett-Teller (BET) method.

### X-ray Photoelectron Spectroscopy (XPS)

Quasi in-situ XPS experiments were carried out in a lab-based NAP-XPS system (SPECS EnviroESCA) with a monochromatic Al Kα x-ray source operated at 50 W. For quasi in-situ XPS experiments, the sample was treated in reaction chamber equipped in the NAP-XPS system with temperature from 250 to 500 °C at 1 bar or with pressures ranging from 1 mbar to 1 bar at 500 °C under a series of gas atmospheres (pure H_2_, pure N_2_, 24%CO_2_/72%H_2_/N_2_, 24%CO_2_/N_2_, 72%H_2_/N_2_, 10%H_2_O/N_2_, 10%CO/N_2_, 10%H_2_O/62%H_2_/N_2_, 10%CO/62%H_2_/N_2_, 10%CO/10%H_2_O/62%H_2_/N_2_). Then, the treated samples were cooled down to room temperature in the gas, evacuated in 1 minute, and transferred to the analysis chamber for XPS measurements. The order of pumping and cooling doesn’t affect the results (Supplementary Fig. 23). For another set of quasi in-situ XPS experiments at 1 bar and 10 bar, the sample after reaction was transferred from an air-tight reaction tube (micro-reactor for atmospheric-pressure or high-pressure reaction) to XPS analysis chamber without exposure to air with the aid of a glovebox and a mobile transfer chamber. The ex*-situ* XPS measurements for the sample taken from the micro-reactor were carried out on a spectrometer equipped with an Al Kα x-ray source operated at 300 W. All spectra were calibrated using the C 1*s* peak at 284.6 eV and all peaks were not normalized.

### Chemical adsorption/Desorption experiments

The temperature-programmed desorption experiments (CO_2_/H_2_/CO-TPD) were performed in a Micromeritics AutoChem II 2920. 0.1 g sample was put into the U tube. For the CO_2_/H_2_-TPD experiments, the fresh samples were pretreated in 75%H_2_/N_2_ at 500 °C for 1 h and then cooled to 50 °C. Then, switch the gas to 5%CO_2_/N_2_ or 10%H_2_/N_2_ for 1 h, and purge the samples for 30 min by helium. After that, the samples were heated from 50 to 800 °C at a rate of 10 °C·min^−1^ under a flow of helium. For the spent samples, the fresh samples were pretreated in 75%H_2_/N_2_ at 500 °C for 1 h and then exposed to 24%CO_2_/72%H_2_/N_2_ at 500 °C for 1 h. The subsequent steps were identical to that of the fresh samples. For the CO-TPD experiments, the steps were identical to CO_2_/H_2_-TPD experiments for the fresh samples, but use 3%CO/N_2_ as the adsorbed gas. The CO_2_/H_2_/CO consumption amounts of catalysts were calculated by TCD signal, which was corrected by the standard Ag_2_O sample.

### Temperature-programmed reaction experiment

The CO_2_ temperature-programmed reaction (CO_2_-TPRe) experiments have been performed on a Micromeritics Autochem 2920 chemisorption analyzer equipped with a Pfeiffer Vacuum OmniStar Mass Spectrometer (MS). Firstly, the sample was reduced under H_2_ at 500 °C for 1 h to remove the surface oxide layer, and then introduced 5%CO_2_/Ar. During the process, MS signals with various mass-to-charge (m/z) ratios were recorded to analyze the gaseous products.

### Theoretical calculations

All the theoretical calculations were carried out by using DFT and ab initio thermodynamics. Details of computational parameters, model constructions, and ab initio thermodynamics are presented in Supplementary Information. With assumption of equilibrium between the gas species under reaction condition, *μ*_o_ and *μ*_c_ were derived by the chemical potentials of the relevant gases, involving CO, CO_2_, H_2_, and H_2_O.

H_2_O shows much higher oxidation ability than CO_2_ confirmed by XPS results in Fig. 2, and thus *μ*_o_ is mainly determined by H_2_O, as shown in Eq. 1.

1 $${\mu }_{{{{{{\rm{O}}}}}}}={\mu }_{{{{{{{\rm{H}}}}}}}_{2}{{{{{\rm{O}}}}}}}-{\mu }_{{{{{{{\rm{H}}}}}}}_{2}}$$

For *μ*_c_ CO dominates the surface carbonization and thus *μ*_c_ is derived as Eq. 2.

2 $${\mu }_{{{{{{\rm{C}}}}}}}={\mu }_{{{{{{\rm{CO}}}}}}}-{\mu }_{{{{{{\rm{O}}}}}}}$$

According to Eq. 3

3 $${\mu }_{{{{{{\rm{gas}}}}}}}={E}_{{{{{{\rm{gas}}}}}}}+ZPE+\delta H-TS+{k}_{B}T\,{{{{\mathrm{ln}}}}}\,\frac{{P}_{gas}}{{P}^{\circ }}$$

The chemical potentials of H_2_O and H_2_ are as below,

4 $${\mu }_{{{{{\rm{H}}}}}_{2}{{{\rm{O}}}}} ={E}_{{{{{\rm{H}}}}}_{2}{{{\rm{O}}}}}+ZP{E}_{{{{{\rm{H}}}}}_{2}{{{\rm{O}}}}}+\delta {H}_{{{{{\rm{H}}}}}_{2}{{{\rm{O}}}}}-T{S}_{{{{{\rm{H}}}}}_{2}{{{\rm{O}}}}}+{k}_{B}T\,{{\mathrm{ln}}}\,\frac{{P}_{{{{{\rm{H}}}}}_{2}{{{\rm{O}}}}}}{{P}^{\circ }}$$

5 $${\mu }_{{{{{\rm{H}}}}}_{2}}={E}_{{{{{\rm{H}}}}}_{2}}+ZP{E}_{{{{{\rm{H}}}}}_{2}}+\delta {H}_{{{{{\rm{H}}}}}_{2}}-T{S}_{{{{{\rm{H}}}}}_{2}}+{k}_{B}T\,{{\mathrm{ln}}}\,\frac{{P}_{{{{{\rm{H}}}}}_{2}}}{{P}^{\circ }}$$

Then, *μ*_o_ is

6 $${\mu }_{{{{\rm{O}}}}} 	= {\mu }_{{{{{\rm{H}}}}}_{2}{{{\rm{O}}}}}-{\mu }_{{{{{\rm{H}}}}}_{2}} \\ 	=\left({E}_{{{{{\rm{H}}}}}_{2}{{{\rm{O}}}}}+ZP{E}_{{{{{\rm{H}}}}}_{2}{{{\rm{O}}}}}+\delta {H}_{{{{{\rm{H}}}}}_{2}{{{\rm{O}}}}}-T{S}_{{{{{\rm{H}}}}}_{2}{{{\rm{O}}}}}+{k}_{B}T\,{{\mathrm{ln}}}\,\frac{{P}_{{{{{\rm{H}}}}}_{2}{{{\rm{O}}}}}}{{P}^{\circ }}\right)\\ 	 \,\,\,\,-\left({E}_{{{{{\rm{H}}}}}_{2}}+ZP{E}_{{{{{\rm{H}}}}}_{2}}+\delta {H}_{{{{{\rm{H}}}}}_{2}}-T{S}_{{{{{\rm{H}}}}}_{2}}+{k}_{B}T\,{{\mathrm{ln}}}\,\frac{{P}_{{{{{\rm{H}}}}}_{2}}}{{P}^{\circ }}\right)\\ 	=({E}_{{{{{\rm{H}}}}}_{2}{{{\rm{O}}}}}+ZP{E}_{{{{{\rm{H}}}}}_{2}{{{\rm{O}}}}}+\delta {H}_{{{{{\rm{H}}}}}_{2}{{{\rm{O}}}}}-T{S}_{{{{{\rm{H}}}}}_{2}{{{\rm{O}}}}})\\ 	 \,\,\,\,-({E}_{{{{{\rm{H}}}}}_{2}}+ZP{E}_{{{{{\rm{H}}}}}_{2}} +\delta {H}_{{{{{\rm{H}}}}}_{2}}-T{S}_{{{{{\rm{H}}}}}_{2}})+{k}_{B}T\,{{\mathrm{ln}}}\,\frac{{P}_{{{{{\rm{H}}}}}_{2}{{{\rm{O}}}}}}{{P}_{{{{{\rm{H}}}}}_{2}}}$$

where *E*_gas_ is the energy of gas phase species by DFT at 0 K. *ZPE* is the zero point energy; δ*H* is the integral of heat capacity; *TS* is the entropic temperature contribution; ${k}_{B}$ is the Boltzmann constant; *T* is the absolute temperature; *P*_gas_ is the partial pressure (Supplementary Table 1); and *P*^o^ is the reference pressure (1 bar). At given *T*, the values of *ZPE*, δ*H*, and *TS* are constant and thus *μ*_o_ is just dependent on the ratio of $P_{{{\mbox{H}}}_{2}{0}}$ to $P_{{{\mbox{H}}}_{2}}$, which is constant. Thus, *μ*_o_ is unchanged with the total reaction pressure at fixing composition. For *μ*_c_ it is the dependent variable of the CO partial pressure at a fixed *μ*_o_ as defined by the Eqs. 2 and 3.

### Supplementary information

Supplementary Information

Peer Review File

## Data Availability

All data that support the findings in this paper are available within the article and its Supporting Information or are available from the corresponding authors upon request. Source data are provided with the paper.
