# Supplementary material for: Reverse water gas-shift reaction product driven dynamic activation of molybdenum nitride catalyst surface
Source: Nat Commun. 2024 Apr 10;15:3100. doi: 10.1038/s41467-024-47550-8 (PMC11271606; doi:10.1038/s41467-024-47550-8)
Supplement: Supplementary file 1 — Supplementary Information [file 41467_2024_47550_MOESM1_ESM.docx]

**Supplementary Information**

**Reverse water gas-shift reaction product driven dynamic activation of molybdenum nitride catalyst surface**

Hui Xin^1,2†^, Rongtan Li^1†^, Le Lin^1^, Rentao Mu^1^, Mingrun Li^1^, Dan Li^3*^, Qiang Fu^1*^, Xinhe Bao^1*^

^1^ State Key Laboratory of Catalysis, Dalian Institute of Chemical Physics, iChEM, Chinese Academy of Sciences, Dalian 116023, China

^2^ Analytical & Testing Center, Sichuan University, Chengdu, Sichuan 610064, China

^3^ Key Laboratory of Green Chemistry and Technology, Ministry of Education, College of Chemistry, Sichuan University, Chengdu, Sichuan 610064, China

^†^ These authors contributed equally: Hui Xin, Rongtan Li.

Emails: [danli@scu.edu.cn](mailto:danli@scu.edu.cn); [qfu@dicp.ac.cn](mailto:qfu@dicp.ac.cn); [xhbao@dicp.ac.cn](mailto:xhbao@dicp.ac.cn).

**Table of Contents**

[Supplementary Methods 4](#_Toc160205990)

[Supplementary Figure 1. The catalytic activity results of fresh β-Mo_2_N catalyst 6](#_Toc160205991)

[Supplementary Figure 2. Schematic representation of the XPS set-up for quasi *in-situ* XPS measurement 7](#_Toc160205992)

[Supplementary Figure 3. The Mo 3*d* spectra of fresh β-Mo_2_N. 8](#_Toc160205993)

[Supplementary Figure 4. Quasi *in-situ* and *ex-situ* C 1*s* XPS results of β-Mo_2_N 9](#_Toc160205994)

[Supplementary Figure 5. *In-situ* NAP-XPS results of β-Mo_2_N. 10](#_Toc160205995)

[Supplementary Figure 6. Quasi *in-situ* XPS results of fresh β-Mo_2_N 11](#_Toc160205996)

[Supplementary Figure 7. The N 1*s*/Mo 3*p* spectra of β-Mo_2_N in atmospheres. 12](#_Toc160205997)

[Supplementary Figure 8. The C 1*s* spectra of β-Mo_2_N from quasi *in-situ* XPS results. 13](#_Toc160205998)

[Supplementary Figure 9. The MS result of β-Mo_2_N upon introducing CO_2_.. 14](#_Toc160205999)

[Supplementary Figure 10. Quasi *in-situ* XPS results of fresh β-Mo_2_N in reaction product atmosphere. 15](#_Toc160206000)

[Supplementary Figure 11. DFT results. 16](#_Toc160206001)

[Supplementary Figure 12. Quasi *in-situ* C 1*s* and N 1*s*/Mo 3*p* spectra of the β-Mo_2_N-1 bar exposed to N_2_. 17](#_Toc160206002)

[Supplementary Figure 13. C 1*s* XPS spectra of β-Mo_2_N catalyst in RWGS reaction with different temperatures 18](#_Toc160206003)

[Supplementary Figure 14. HRTEM image of the β-Mo_2_N catalysts 19](#_Toc160206004)

[Supplementary Figure 15. Quasi *in-situ* XPS results of the treated β-Mo_2_N samples. .20](#_Toc160206005)

[Supplementary Figure 16. XRD patterns of the MoC and Mo_2_N materials. 21](#_Toc160206006)

[Supplementary Figure 17. The catalytic activity results of β-Mo_2_N-1bar catalyst. 22](#_Toc160206007)

[Supplementary Figure 18. The catalytic activity results of α-MoC catalyst 23](#_Toc160206008)

[Supplementary Figure 19. Quasi *in-situ* XPS results of γ-Mo_2_N catalyst 24](#_Toc160206009)

[Supplementary Figure 20. The catalytic activity results of fresh γ-Mo_2_N and γ-Mo_2_N-1bar catalysts 25](#_Toc160206010)

[Supplementary Figure 21. The C/Mo surface atom ratios of fresh β-Mo_2_N and γ-Mo_2_N. 26](#_Toc160206011)

[Supplementary Figure 22. CO temperature programmed desorption analysis. 27](#_Toc160206012)

[Supplementary Figure 23. Quasi *in-situ* XPS results of β-Mo_2_N with different procedures.](#_Toc160206013) 28

[Supplementary Table 1. Assumed pressure (*P*) equilibrium conditions. 29](#_Toc160206014)

[Supplementary Table 2. Physiochemical properties. 30](#_Toc160206015)

[Supplementary Table 3. CO desorption amount.. 31](#_Toc160206016)

[Supplementary Table 4. DFT energy correction data.. 32](#_Toc160206017)

[Supplementary Table 5. DFT data for derivation of the O_2_ and graphite C energies. 33](#_Toc160206018)

[Supplementary Table 6. Computational setting. 34](#_Toc160206019)

[Supplementary Table 7. DFT energy data for the gases. 35](#_Toc160206020)

[Supplementary References 36](#_Toc160206021)

# Supplementary Methods

1. **Computational parameters**

Spin-polarized DFT calculations were implemented using a plane-wave basis set in the Vienna Ab-initio Simulation Packages (VASP 5.4)^1^. The exchange-correlation energy was treated using Perdew-Burke-Ernzerhof (PBE) functional within the generalized gradient approximation (GGA)^2^. The projected-augmented wave (PAW) pseudopotentials were utilized to describe the core electrons, and a cutoff energy of 400 eV was used for the plane-wave expansion^3^. The following valence electron configurations were included in the self-consistent field calculations: Mo (4d^5^ and 5s^1^), O (2s^2^ and 2p^4^), and C (2s^2^ and 2p^2^). In addition, the van der Waals (vdW) dispersion forces were corrected by the vdW-DF (optPBE) function, which showed highly accurate description for oxides^4,5^. An on-site Hubbard term U_eff_ = U – J was added to address the open-shell *d*-electrons with 3.23 eV for oxidative Mo (i.e., 2MoO_2_ + O_2_ = 2MoO_3_) while with no correction on metallic and carbonized Mo for which an arbitrary correction is used with Δ*E*_M_ = 2.65 eV/Mo for Mo + O_2_ = MoO_2_, Δ*E*_M_ = -0.02 eV/Mo for Mo + 2C = Mo_2_C, and Δ*E*_M_ = 0.45 eV/Mo for Mo + C = MoC, respectively to align to the experimental formation enthalpies (Supplementary Table 4)^6-8^. The water-based reference state for the calculations to avoid incorrect description of the gas phase O_2_ reference with standard DFT methods (Supplementary Table 5)^4^. The energies and residual forces were converged to 10^-5^ eV and 0.02 eV Å^-1^, respectively.

1. **Model constructions**

To understand the transformation between the experimental MoO*_x_* and MoC*_x_* species on β-Mo_2_N surfaces, we adopt five Mo compounds as prototypes to compare the thermodynamics of various MoO*_x_* and MoC*_x_* phases, including metallic Mo, MoO_2_, MoO_3_, Mo_2_C, and MoC crystals. The details of the structures and the computational settings are shown in Supplementary Table 6 and Supplementary Figure 11.

1. **Ab initio thermodynamics**
2. The phase diagram is based on the ab initio thermodynamic analysis by which we can locate the phase at given conditions. The formation Gibbs free energy (*ΔG*_form_) was defined as

$\Delta G_{\text{form}}=E_{\text{Mo}_{x}\text{O}_{y}\text{C}_{z}}-xE_{\text{Mo}}-yE_{\text{O}}-zE_{\text{C}}$ (1)

Where E_Mo_*_x_*_O_*_y_*_C_*_z_* and *E*_Mo_ are the electric energies of bulk Mo compounds and metallic Mo by DFT at 0 K, respectively.

1. The chemical potentials of solid species (*G*_solid_) were calculated by

$G_{\text{solid}}=E_{\text{solid}}+ZPE+\delta H-TS$ (2)

where *E*_solid_ is the energy of solid phase species by DFT at 0 K. *ZPE* is the zero point energy; δ*H* is the integral of heat capacity; *TS* is the entropic temperature contribution;

For gaseous species (*μ*_gas_), the corrections were taken from the *NIST* database through standard ideal-gas method^9,10^. For solid phase species (*G*_solid_), the last three items were obtained by vibrational frequencies calculations via standard methods^9,10^. The corrected values were listed in Supplementary Table 7 via VASPKIT^11^.

Supplementary figures


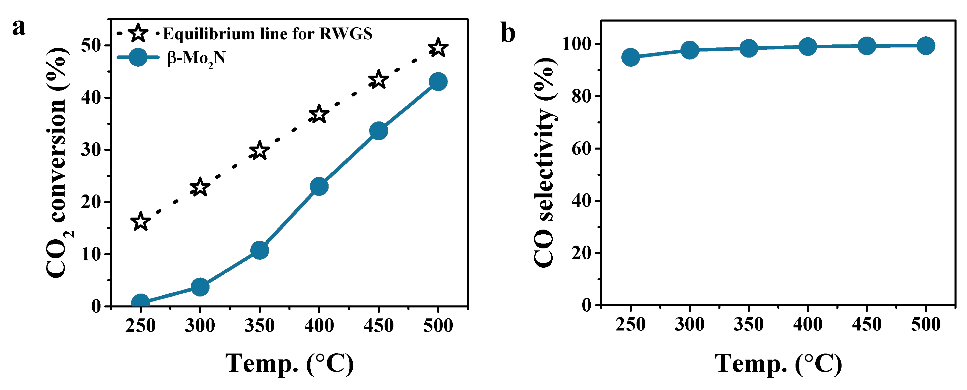


# Supplementary Figure 1. The catalytic activity results of fresh β-Mo_2_N catalyst. (a) CO_2_ conversion. (b) CO selectivity. Reaction condition: 50 mg catalyst, 1 bar 24%CO_2_/72%H_2_/N_2_, WHSV = 30,000 mL⋅g_catal_^-1^⋅h^-1^.


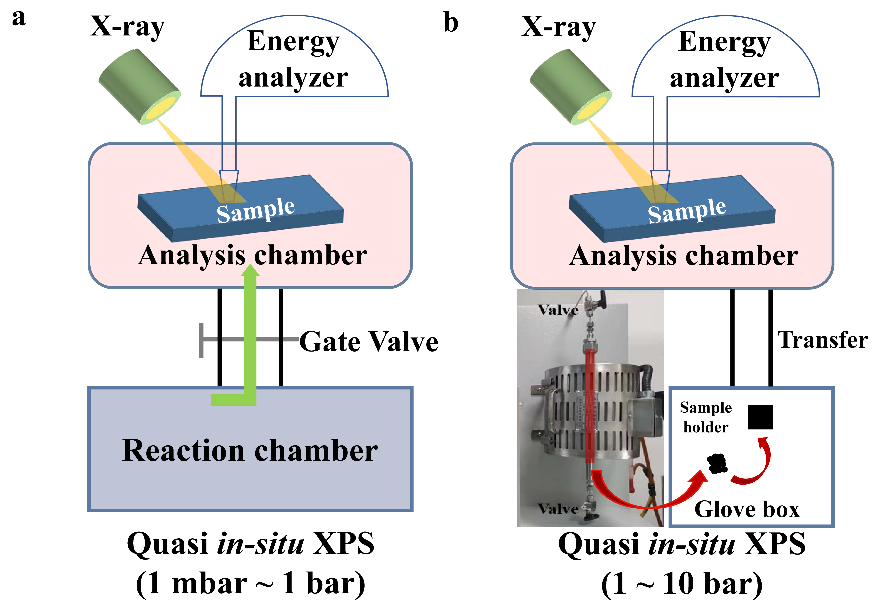


# Supplementary Figure 2. Schematic representation of the XPS set-up for quasi *in-situ* XPS measurement. (a) 1 mbar to 1 bar, (b) 1 to 10 bar.


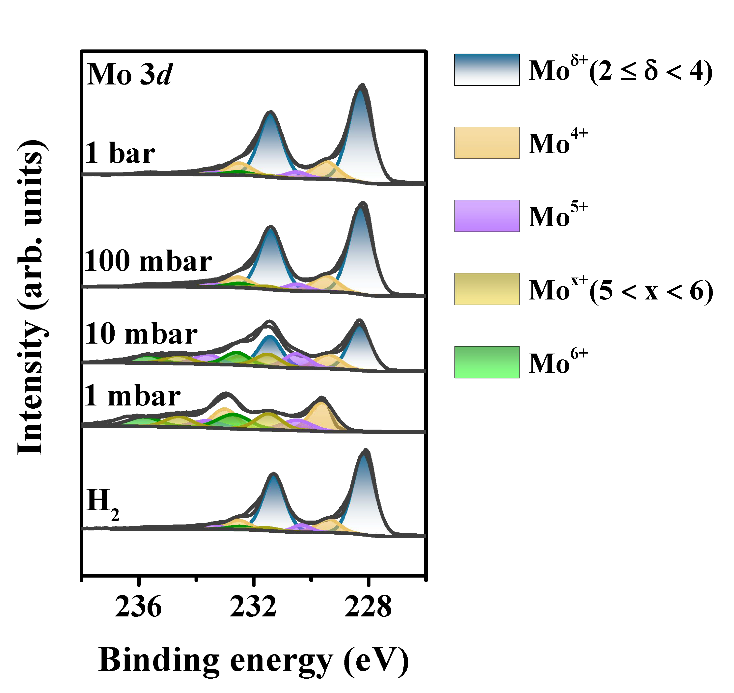


# Supplementary Figure 3. The Mo 3*d* spectra of fresh β-Mo_2_N. Measured condition is under 24%CO_2_/72%H_2_/N_2_ from 1 mbar to 1 bar at 500 ℃ by quasi *in-situ* XPS.

Each surface component can be assigned to the following species: Mo^δ+^ (2 ≤ δ < 4) in Mo_2_N at 228.2 eV, Mo^4+^ at 229.5 eV in MoO_2_, Mo^5+^ species at 230.5 eV, Mo^x+^ (5 < x < 6) and Mo^6+^ in MoO_3_ at 232.5 eV^12-15^.





# Supplementary Figure 4. Quasi *in-situ* and *ex-situ* C 1*s* XPS results of β-Mo_2_N sample. The *ex-situ* XPS results is taken from the micro-reactor after reaction under 1 bar 24%CO_2_/72%H_2_/N_2_ at 500 ℃ for 1 h and subsequent exposure to air for 5 minutes and 1 day, respectively.


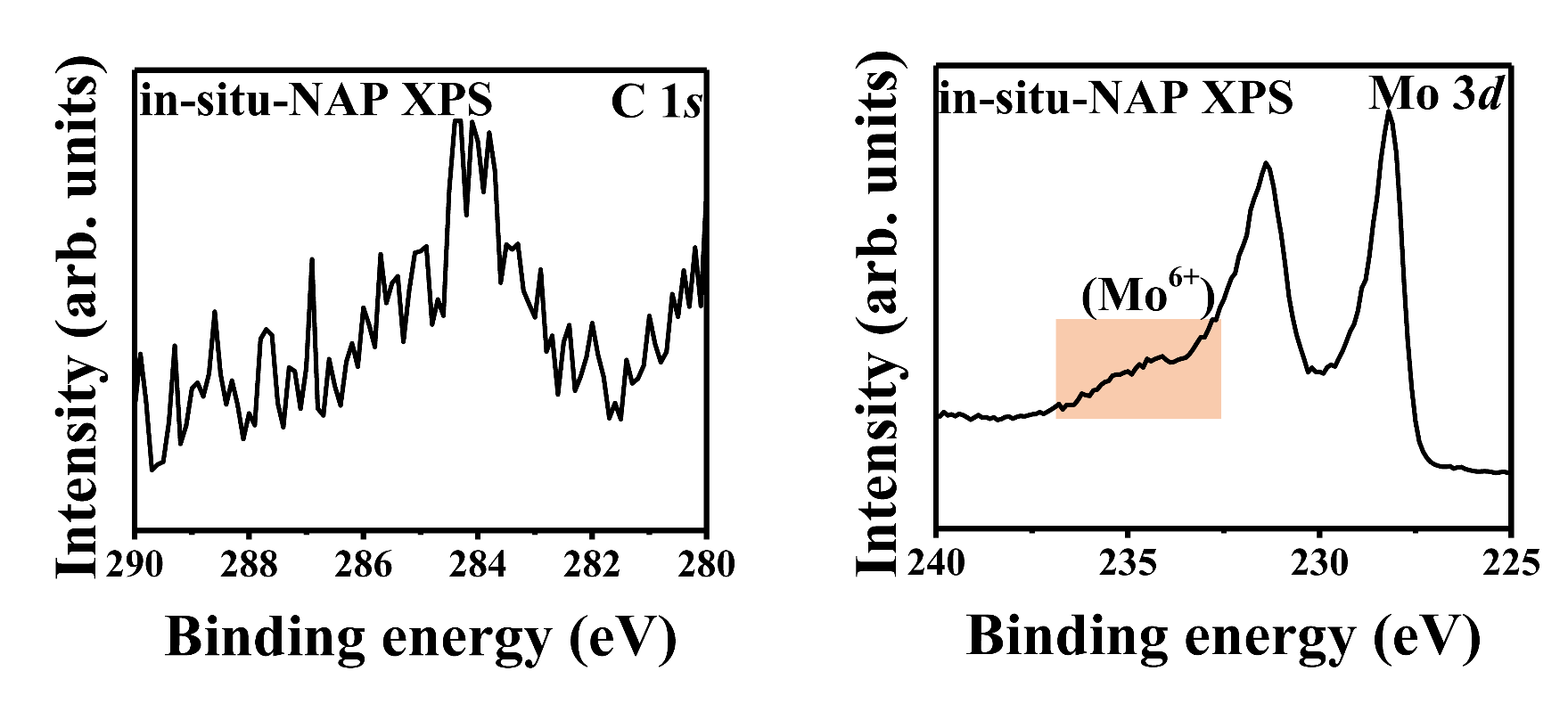


# Supplementary Figure 5. *In-situ* NAP-XPS results of β-Mo_2_N. C 1*s* and Mo 3*d* of β-Mo_2_N under 1 mbar 24%CO_2_/72%H_2_/N_2_, at 500 ℃ showing the presence of surface MoO*_x_* but not surface MoC*_x_* species.


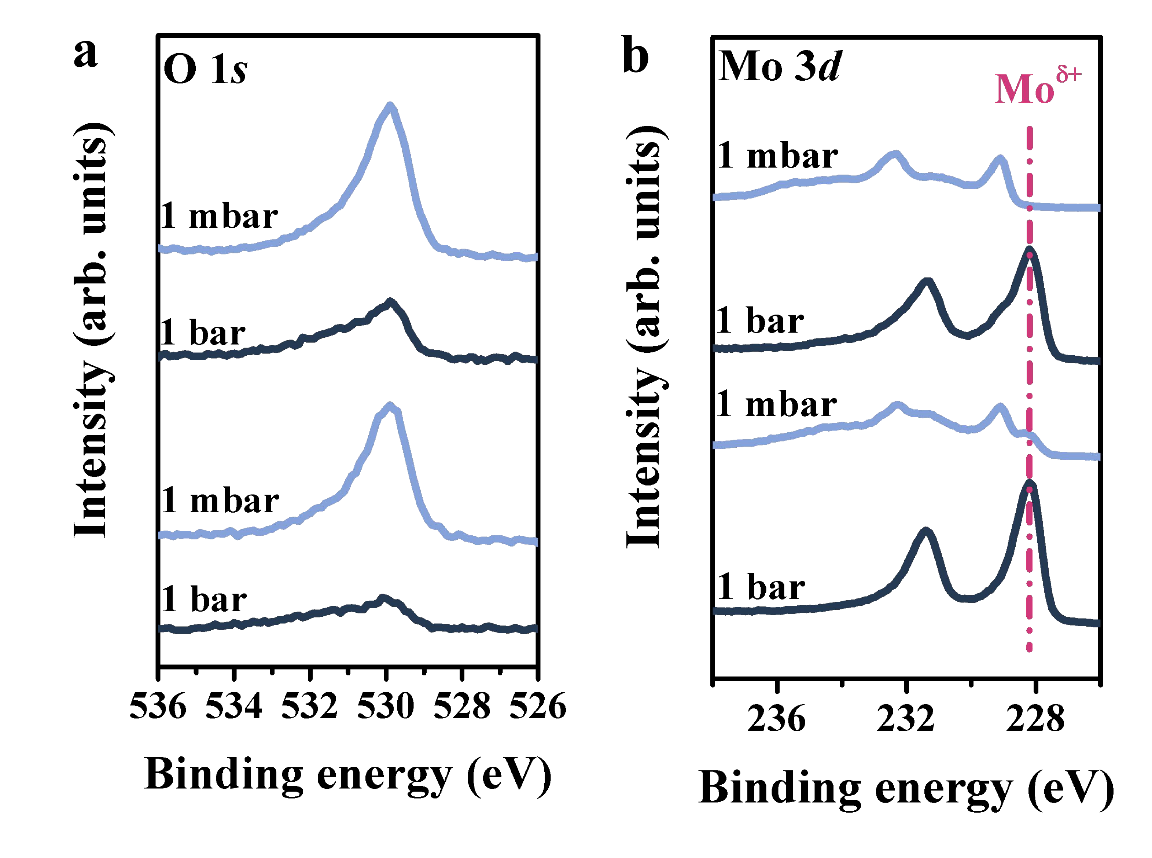


# Supplementary Figure 6. Quasi *in-situ* XPS results of fresh β-Mo_2_N. (a) O 1*s* and (b) Mo 3*d* spectra. The loop experiments were conducted with 1 mbar and 1 bar 24%CO_2_/72%H_2_/N_2_ at 500 ℃.





# Supplementary Figure 7. The N 1*s*/Mo 3*p* spectra of β-Mo_2_N in different atmospheres. The pre-reduced β-Mo_2_N is exposed to 1 bar 72%H_2_/N_2_, 1 bar 24%CO_2_/N_2_, 1 bar 10%H_2_O/N_2_, and 1 bar 10%CO/N_2_, respectively at 500 °C for 30 minutes.





# Supplementary Figure 8. The C 1*s* spectra of β-Mo_2_N from quasi *in-situ* XPS results. The sample is treated by 1 bar 10%CO/N_2_ and then switched to 1 bar 72%H_2_/N_2_ at 500 ℃.





# Supplementary Figure 9. The MS result of β-Mo_2_N upon introducing CO_2_. The β-Mo_2_N is pre-reducing under H_2_ at 500 ℃ for 1 h, and then introducing 5%CO_2_/Ar. The MS signal of CO is deducted the CO fragment ion signal from CO_2_.





# Supplementary Figure 10. Quasi *in-situ* XPS results of fresh β-Mo_2_N in reaction product atmosphere. The O 1*s* spectra measured under 1 mbar and 1 bar CO/H_2_/N_2_ and CO/H_2_O/H_2_/N_2_ atmospheres, respectively. The ratio of H_2_ is 62%, CO and H_2_O in mixed atmospheres are all 10%, similar to that in real reaction environment at 500 ℃.


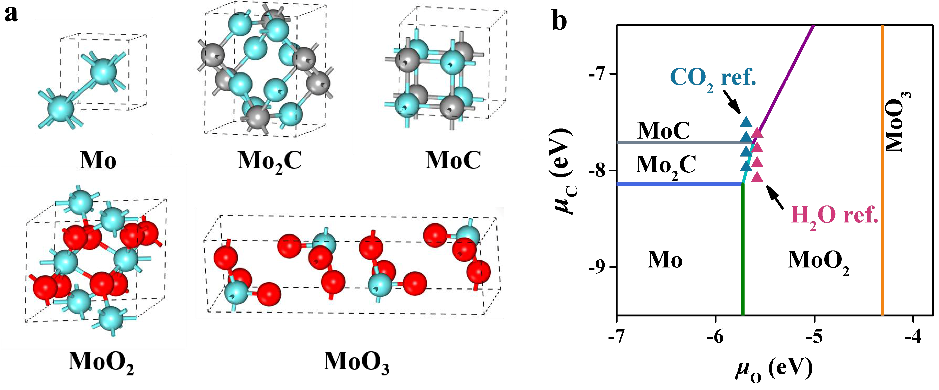


# Supplementary Figure 11. DFT results. (a) Unit cells of Mo compounds including metallic Mo, Mo_2_C, MoC, MoO_2_, and MoO_3_ bulks. C: gray; O: red; Mo: cyan. (b) The computed phase diagram of Mo metal, carbides, and oxides. Each solid line denotes the boundary between two domains (phase equilibrium). The Mo species with the lowest free energy is marked for each domain. The triangles represent the position of *μ*_o_ and *μ*_c_ within specific reaction equilibrium conditions (Supplementary Table 1).


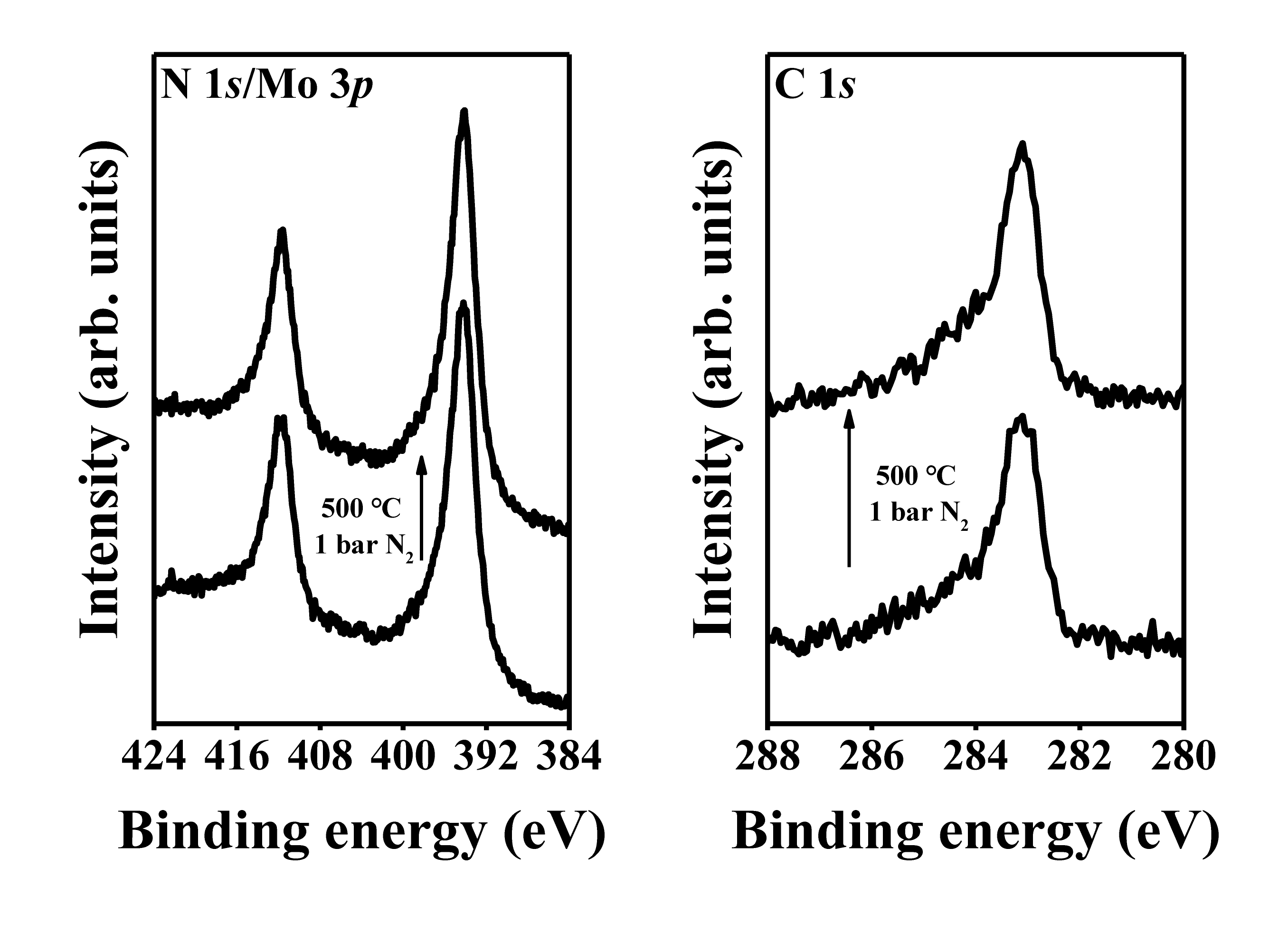


# Supplementary Figure 12. Quasi *in-situ* C 1*s* and N 1*s*/Mo 3*p* spectra of the β-Mo_2_N-1bar exposed to N_2_. The β-Mo_2_N-1bar sample was exposed to 1 bar N_2_ at 500 °C for 30 minutes.


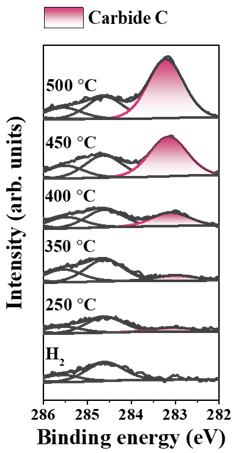


# Supplementary Figure 13. C 1*s* XPS spectra of β-Mo_2_N catalyst in RWGS reaction with different temperatures. The sample were treated at 1 bar 24%CO_2_/72%H_2_/N_2_ and measured by quasi *in-situ* XPS.

#


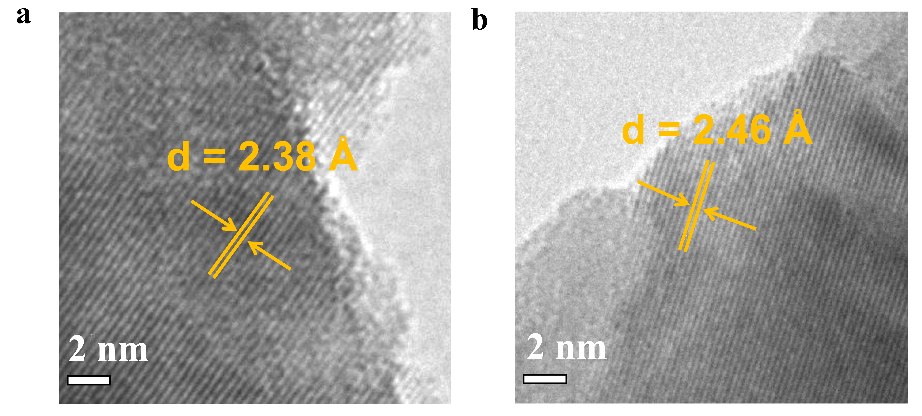


# Supplementary Figure 14. HRTEM image of the β-Mo_2_N catalysts. (a) Fresh β-Mo_2_N and (b) β-Mo_2_N-1bar catalysts.


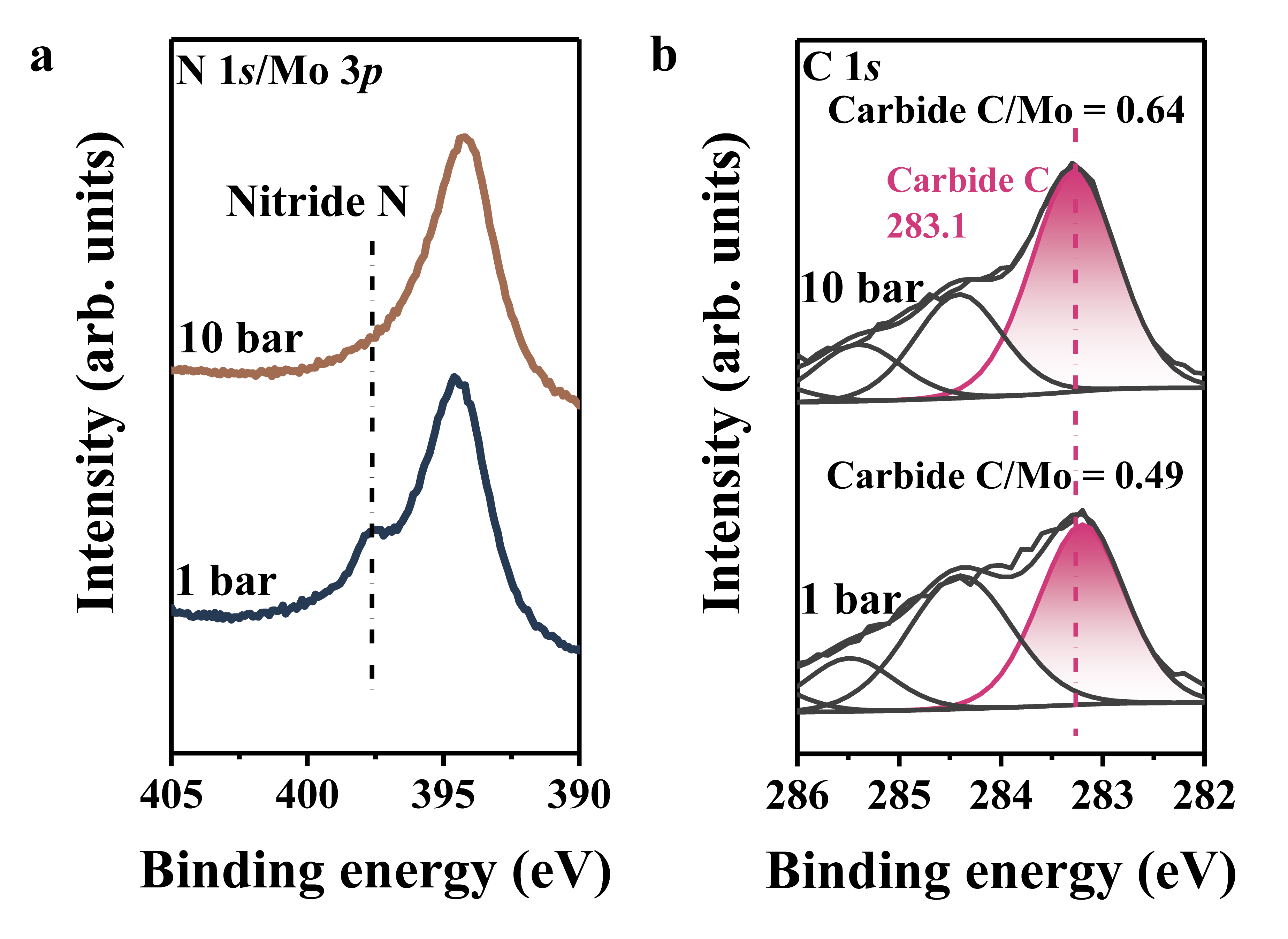


# Supplementary Figure 15. Quasi *in-situ* XPS results of the treated β-Mo_2_N samples. They are taking from the micro-reactor after reaction under 500 ℃, 24%CO_2_/72%H_2_/N_2_ at 1 and 10 bar for 1 h, respectively. (a) N 1*s*/Mo 3*p* and (b) C 1*s* spectra. The surface carbide C/Mo atom ratio is calculated from the ratio of carbide C peak and whole Mo 3*d* spectra, which are listed in Supplementary Figure 14 (b).

The carbide C peak at 283.1 eV is observed on β-Mo_2_N when measured at both 1 and 10 bar. While the nitride N peak on β-Mo_2_N disappeared when treated at 10 bar compared with that treated at 1 bar. Moreover, the surface carbide C/Mo atom ratio at 10 bar condition is 0.64, which is higher that of 1 bar (0.49). These all imply that the stronger carbonization occurs at 10 bar compared with 1 bar condition.


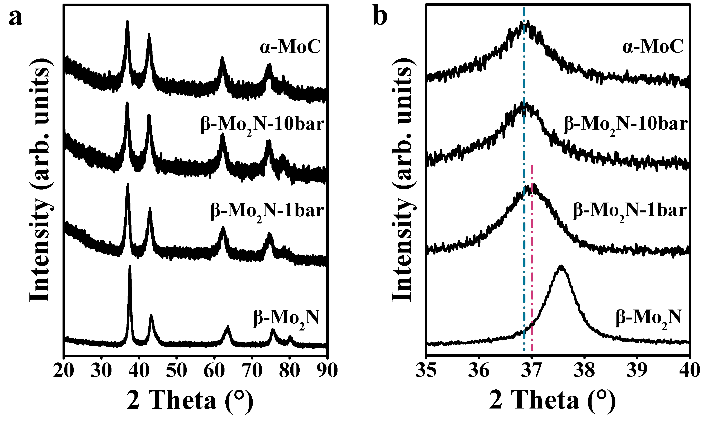


# Supplementary Figure 16. XRD patterns of the MoC and Mo_2_N materials. (a) XRD pattern of fresh α-MoC, fresh β-Mo_2_N and β-Mo_2_N treated in the micro-reactor after reaction under 500 ℃, 24%CO_2_/72%H_2_/N_2_ at 1 and 10 bar for 1 h, respectively; (b) Enlarged area in (a) from 35 - 40 °. The blue and red lines indicate the diffraction peaks of α-MoC and β-Mo_2_N-1 bar, respectively.

**
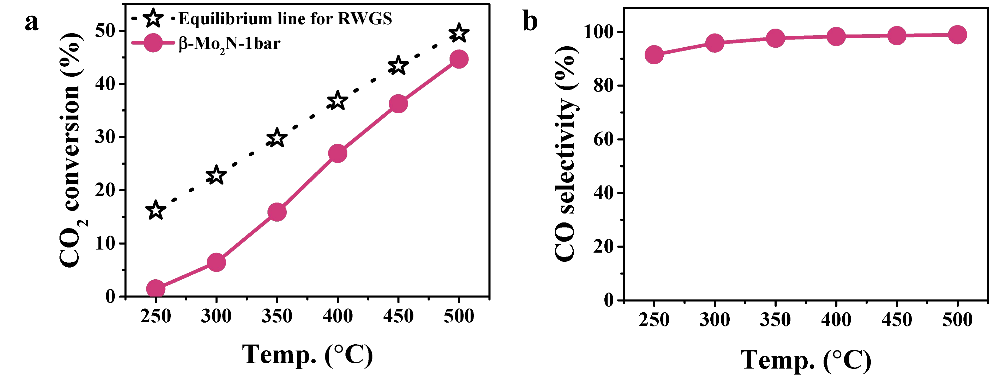
**

# Supplementary Figure 17. The catalytic activity results of β-Mo_2_N-1bar catalyst. (a) CO_2_ conversion. (b) CO selectivity. Reacion condition: 50 mg catalyst, 1 bar 24%CO_2_/72%H_2_/N_2_, WHSV = 30,000 mL⋅g_catal_^-1^⋅h^-1^.


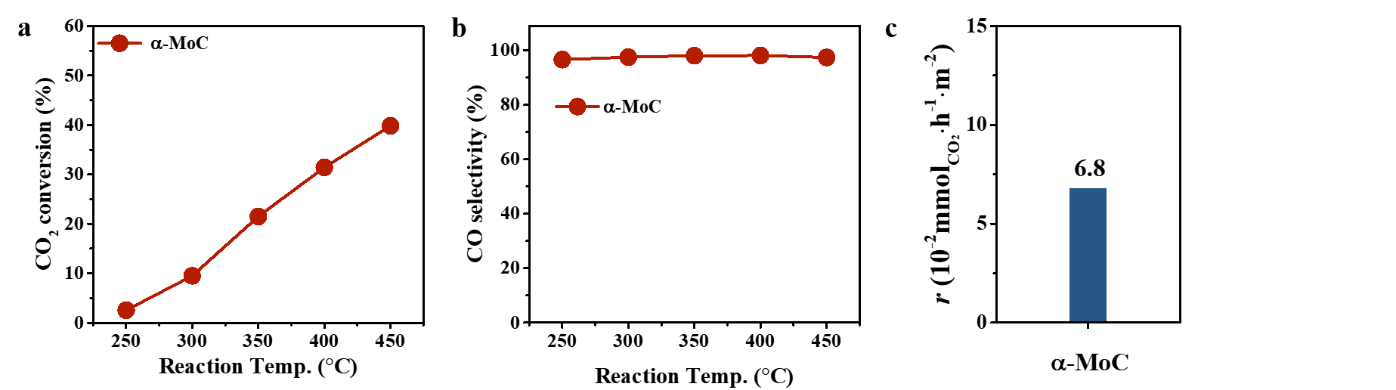


# Supplementary Figure 18. The catalytic activity results of α-MoC catalyst. (a) CO_2_ conversion. (b) CO selectivity. Reaction condition: 50 mg catalyst, 1 bar 24%CO_2_/72%H_2_/N_2_, WHSV = 30,000 mL⋅g_catal_^-1^⋅h^-1^. (c) Reaction rate of CO_2_ conversion on α-MoC catalyst normalized by the specific surface area under reaction condition of 50 mg catalysts, 250 °C, 24%CO_2_/72%H_2_/N_2_, and WHSV = 30,000 mL⋅g_catal_^-1^⋅h^-1^ with the CO_2_ conversion below 10%.


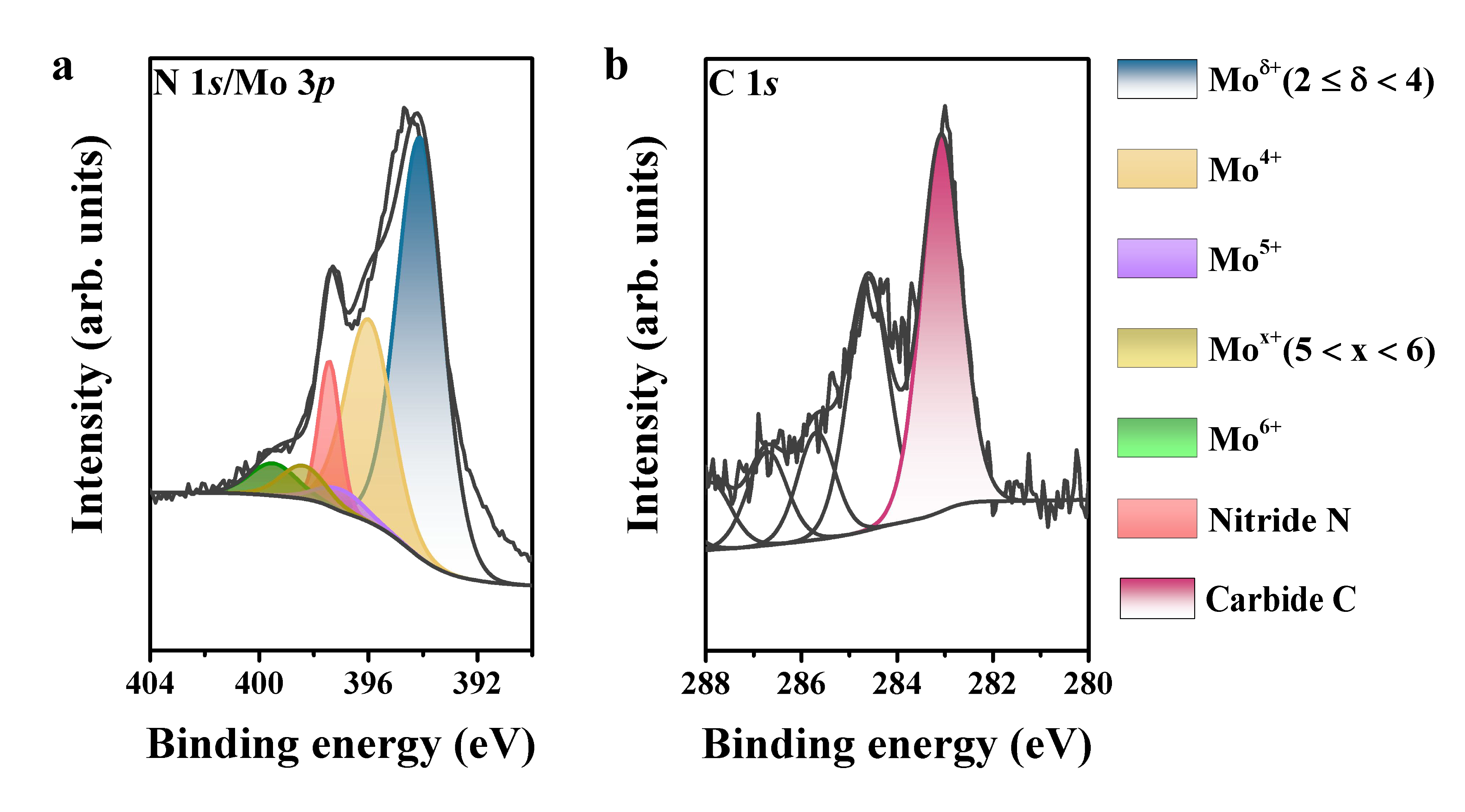


# Supplementary Figure 19. Quasi *in-situ* XPS results of γ-Mo_2_N catalyst. (a) The N 1*s*/Mo 3*p* spectra and (b) C 1*s* spectra, which are measured after pre-reduced under 1 bar H_2_ at 500 ℃ and then treating under 1 bar 24%CO_2_/72%H_2_/N_2_ at 500 ℃ for 1 h.

Under the tested reaction conditions, the carbide C peak at 283.1 eV appears^14^. And its intensity gradually become strengthened along with the weak intensity of nitride N peak at 397.6 eV throughout the reaction^15^. These suggest that the carbonization process happens on the γ-Mo_2_N.


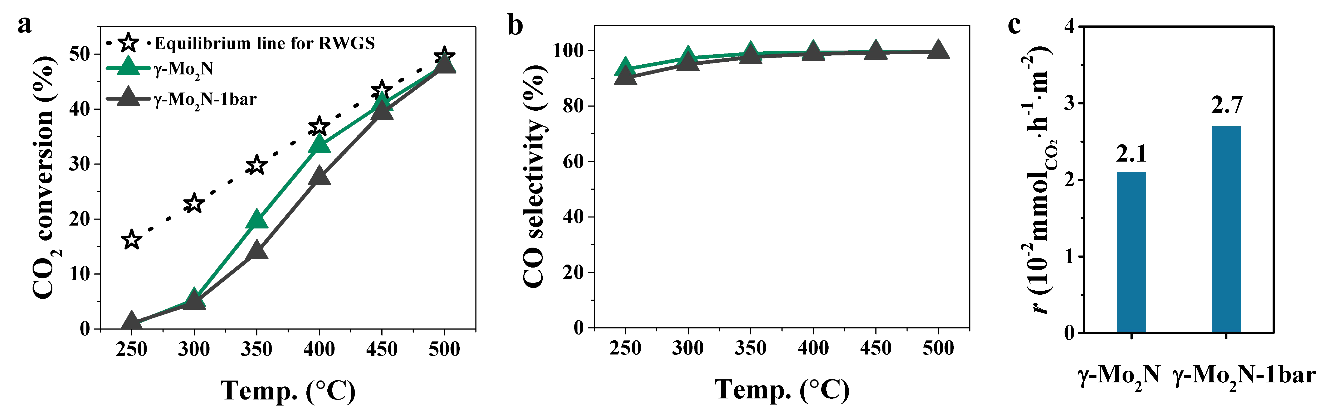


# Supplementary Figure 20. The catalytic activity results of fresh γ-Mo_2_N and γ-Mo_2_N-1bar catalysts. (a) CO_2_ conversion. (b) CO selectivity. Reacion condition: 50 mg catalyst, 1 bar 24%CO_2_/72%H_2_/N_2_, WHSV = 30,000 mL⋅g_catal_^-1^.⋅h^-1^. (c) The reaction rate of CO_2_ conversion on the γ-Mo_2_N catalysts normalized by the specific surface area under reaction condition of 50 mg catalysts, 250 ℃, 24%CO_2_/72%H_2_/N_2_, and WHSV = 30,000 mL⋅g_catal_^-1^⋅h^-1^ with the CO_2_ conversion below 5%.

The fresh γ-Mo_2_N and γ-Mo_2_N-1bar catalysts all display RWGS reactivity from 250 to 500 ℃. They have similar reaction rate at 250 ℃, suggesting that the carbonization has weak effect on the catalytic performance for γ-Mo_2_N-1bar catalyst.





# Supplementary Figure 21. The C/Mo surface atom ratios of fresh β-Mo_2_N and γ-Mo_2_N. They are calculated from Mo 3*d* and C 1*s* spectra of the two samples from the quasi *in-situ* XPS results under condition of 1 bar 24%CO_2_/72%H_2_/N_2_ at 500 ℃.

The C/Mo surface atom ratio is 1.03 on β-Mo_2_N (Fig. 1) while 0.48 on γ-Mo_2_N at the reaction temperature of 500 ℃, respectively. This illustrates that the β-Mo_2_N is much easier to be carbonized and shows deeper carbonization compared with γ-Mo_2_N under the identical reaction conditions.





# Supplementary Figure 22. CO temperature programmed desorption analysis. CO desorption of Fresh β-Mo_2_N and γ-Mo_2_N.

There are two types of adsorption peaks, that is, 85 ~ 112 ℃ and 245 ~ 255 ℃, which are ascribed to physically and chemically adsorbed CO, respectively ^16^. Thus, γ-Mo_2_N shows strong physically adsorbed CO, whereas the chemically adsorbed CO mainly occurs on β-Mo_2_N. This manifests that β-Mo_2_N performs stronger interaction with CO than that on γ-Mo_2_N.


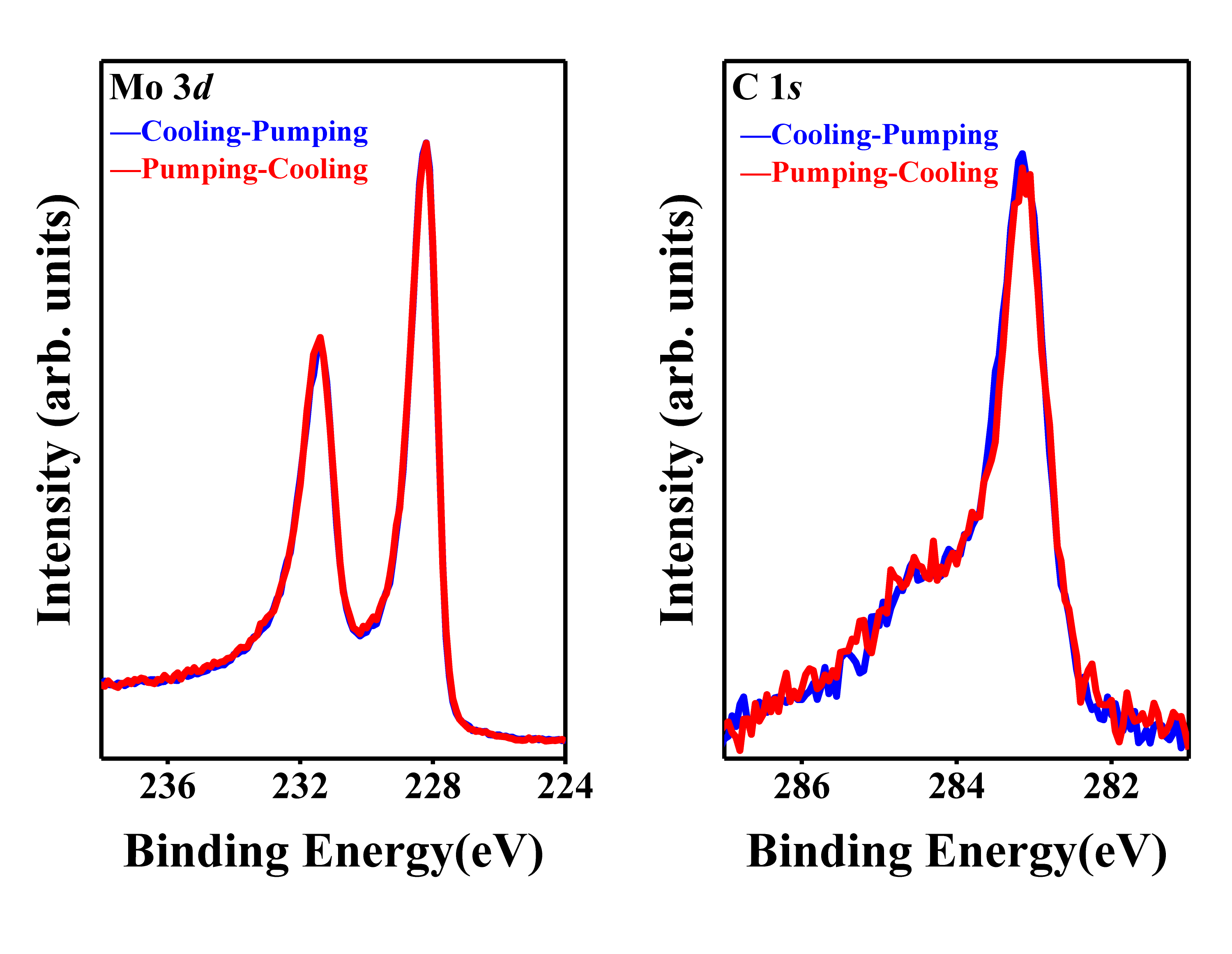


# Supplementary Figure 23. Quasi *in-situ* XPS results of β-Mo_2_N with different procedures. Cooling-Pumping: First cooling in reaction gases and then pumping to high vacuum; Pumping-Cooling: First pumping to high vacuum and then cooling in vacuum. Reaction gas: 1 bar, 24%CO_2_/72%H_2_/N_2_. T = 500 ℃. Supplementary tables

# Supplementary Table 1. Assumed pressure (*P*) equilibrium conditions. The assumption is based on the 43.1% CO_2_ conversion considered starting from a feed reactants ratio of 24%CO_2_/72%H_2_/N_2_.

| Total *P* | *P*_CO2_ | *P*_H2_ | *P*_CO_ | *P*_H2O_ |
| --- | --- | --- | --- | --- |
| mbar | mbar | mbar | mbar | mbar |
| 1 | 0.14 | 0.62 | 0.1 | 0.1 |
| 10 | 1.4 | 6.2 | 1 | 1 |
| 100 | 14 | 62 | 10 | 10 |
| 1000 | 140 | 620 | 100 | 100 |

# Supplementary Table 2. Physiochemical properties. BET surface area of β-Mo_2_N, β-Mo_2_N-1 bar and α-MoC catalysts.

| Samples | BET surface area (m^2^/g) |
| --- | --- |
| β-Mo_2_N | 21.5 |
| β-Mo_2_N-1bar | 17.1 |
| α-MoC | 108.7 |

# Supplementary Table 3. CO desorption amount. The desorption amount of CO on the fresh γ-Mo_2_N and β-Mo_2_N catalysts.

| Catalysts | CO desorption (μmol/m^2^) ^[a]^ | |
| --- | --- | --- |
|  | 85 ~ 112 ℃ | 245 ~ 255 ℃ |
| γ-Mo_2_N | 1.87 | 0.11 |
| β-Mo_2_N | 0.66 | 1.39 |

^[a]^ Calculated based on the CO-TPD profiles

The β-Mo_2_N shows higher chemically adsorbed CO amount than that on γ-Mo_2_N. This explains why the β-Mo_2_N performs deeper carbonization than that on γ-Mo_2_N under the identical reaction conditions.

# Supplementary Table 4. DFT energy correction data. Data for derivation of arbitrary corrections (Δ*E*_M_) on metallic and carbonized Mo after Hubbard U corrections on oxidative Mo.

| Species | GGA energy ^[a]^ | U_eff_ | GGA+U energy ^[b]^ | Δ_rxn_*H*_exp_@298 K ^[c]^ |
| --- | --- | --- | --- | --- |
|  | eV | eV | eV | eV |
| Mo | -8.12 | -- | -- | -- |
| MoO_2_ | -20.40 | 3.23 | -16.92 | -6.09 |
| MoO_3_ | -25.07 | 3.23 | -21.23 | -7.72 |
| Mo_2_C | -24.43 | -- | -- | -0.51 |
| MoC | -15.60 | -- | -- | -0.29 |
| O_2_ | -5.36 ^[d]^ | -- | -- | -- |
| C | -7.74 ^[e]^ | -- | -- | -- |

^[a]^ Energy per stoichiometry unit based on the vdW-DF (optPBE) functional.

^[b]^ Energy per stoichiometry unit after U correction.

^[c]^ Data from the reference ^17^.

^[d]^ Energy is derived from the water-reference scheme through preserving the reaction energy of H_2_ (g) + 0.5O_2_ (g) → H_2_O (g) (Δ_rxn_*H*_exp_@298 K = -2.51 eV) ^4^. See Supplementary Table 5.

^[e]^ Energy is corrected by preserving the reaction energy of 2H_2_ (g) + C (s) → CH_4_ (g) (Δ_rxn_*H*_exp_@298 K = -0.78 eV). See Supplementary Table 5.

# Supplementary Table 5. DFT data for derivation of the O_2_ and graphite C energies. It includes DFT energy, zero-point energy (*ZPE*), integrated heat capacity (δ*H*), and standard formation enthalpies at 298 K (Δ_rxn_*H*_exp_@298 K) for the relevant gases and solids. ^[a]^ (Unit: eV)

| Gas | GGA energy | *ZPE* ^[b]^ | δ*H* ^[b]^ | Δ_rxn_*H*_exp_@298 K |
| --- | --- | --- | --- | --- |
|  | eV | eV | eV | eV |
| H_2_ | -6.80 | 0.27 | 0.09 | -- |
| H_2_O | -12.21 | 0.57 | 0.57 | -2.51 |
| CH_4_ | -25.07 | 1.19 | 0.10 | -0.78 |
| C* in graphite | -- | 0.25 | 0.00 | -- |
| C* in Mo_2_C | -- | 0.09 | 0.02 | -- |
| C* in MoC | -- | 0.13 | 0.01 | -- |

^[a]^ Note that the thermodynamic contributions of Mo in solids can be neglected because they would be effectively cancelled out in the reaction. And the *ZPE*+δ*H* of O can be cancelled out and neglected when transferring from gas phase O_2_ to solid phase O* as reported ^4^.

^[b]^ For gas phase species, the δ*H* is available in the standard Supplementary Tables ^18^. For solid species, we calculated the corresponding data using phonon by standard methods ^9^.

# Supplementary Table 6. Computational setting. Detailed computational setting on the bulk crystals.^[a]^

| Species | Space group | Lattice parameters (Å) | | | Lattice angle (°) | | | K-points | Magnetics ^[b]^ |
| --- | --- | --- | --- | --- | --- | --- | --- | --- | --- |
|  |  | *a* | *b* | *c* | *α* | *β* | *γ* |  |  |
| Mo | Im$\bar{3}$m | 6.32 | 6.32 | 6.32 | 90 | 90 | 90 | 7×7×7 | NM |
| MoO_2_ | P2_1_/c | 5.96 | 4.81 | 5.78 | 90 | 119.77 | 90 | 5×5×5 | AFM |
| MoO_3_ | Pnma | 3.75 | 3.97 | 14.36 | 90 | 90 | 90 | 7×6×2 | NM |
| Mo_2_C | Pbcn | 4.76 | 6.08 | 5.26 | 90 | 90 | 90 | 5×4×5 | NM |
| MoC | Pm$\bar{3}$m | 4.40 | 4.40 | 4.40 | 90 | 90 | 90 | 6×6×6 | NM |

^[a]^ Mo and its carbides are shown without U correction. Mo oxides are with U_eff_ = 3.23 eV correction.

^[b]^ NM: Non-magnetic; AFM: Anti-ferromagnetic; FM: Ferromagnetic.

# Supplementary Table 7. DFT energy data for gases. DFT energy, Zero-point energy (*ZPE*), integrated heat capacity (δ*H*), entropic temperature correction, and total Gibbs free energy corrections (*G-E*_elec_) for the gases. (*T* = 500 ℃)

| Species | *E*_elec_ ^[a]^ | *ZPE* | δ*H* | *TS* | *G-E*_elec_ ^[b]^ |
| --- | --- | --- | --- | --- | --- |
|  | eV | eV | eV | eV | eV |
| CO | -11.65 ^[a]^ | 0.13 | 0.23 | 1.90 | -1.54 |
| CO_2_ | -17.76 ^[a]^ | 0.31 | 0.29 | 2.14 | -1.54 |
| H_2_ | -6.80 | 0.27 | 0.23 | 1.31 | -0.81 |
| H_2_O | -12.21 | 0.57 | 0.27 | 1.82 | -0.98 |

^[a]^ For CO and CO_2_ molecules, there are errors on their energies within the vdW-DF (optPBE) functional calculation and the corrections are necessary with the sensitivity analysis reported ^10,19^. Here, a correction of +0.14 eV was made to the CO energy while +0.54 eV is for CO_2_ through minimizing the root mean square deviation (RMSD = 0.06 eV) for alignment to the experimental reaction enthalpies.

^[b]^ For high temperature (500 ℃) conditions, considering the thermodynamic corrections of the gas phase species is enough because the correction (*G-E*_elec_) on solid species is negligible with the order of magnitude of ~0.01 eV.

# Supplementary References

1. Kresse, G. & Furthmüller, J. Efficiency of ab-initio total energy calculations for metals and semiconductors using a plane-wave basis set. *Comp. Mater. Sci.* **6,** 15-50 (1996).

2. Perdew, J. P., Burke, K. & Ernzerhof, M. Generalized gradient approximation made simple. *Phys. Rev. Lett.* **77,** 3865-3868 (1996).

3. Kresse, G. & Joubert, D. From ultrasoft pseudopotentials to the projector augmented-wave method. *Phys. Rev. B*. **59,** 1758-1775 (1999).

4.Zeng, Z. et al. Towards first principles-based prediction of highly accurate electrochemical pourbaix diagrams. *J. Phys. Chem. C*. **119,** 18177-18187 (2015).

5. Lin, L., Zeng, Z., Fu, Q. & Bao, X. Achieve flexible large-scale reactivity tuning by controlling phase, thickness and support of two-dimensional ZnO. *Chem. Sci.* **12,** 15284-15290 (2021).

6. Zeng, Z. et al. Stabilization of ultrathin (hydroxy)oxide films on transition metal substrates for electrochemical energy conversion. *Nat. Energy*. **2,** 17070 (2017).

7. Li, Y. et al. BEEF-vdw+U method applied to perovskites: Thermodynamic, structural, electronic, and magnetic properties. *J. Phys.: Condens. Matter*. **31,** 145901 (2019).

8. Zhu, J. et al. Dynamic structural evolution of iron catalysts involving competitive oxidation and carburization during CO_2_ hydrogenation. *Sci. Adv.* **8,** eabm3629 (2022).

9. Cramer, C. J. Essentials of computational chemistry: theories and models. *John Wiley & Sons*. (2013).

10. Peterson, A. A. et al. How copper catalyzes the electroreduction of carbon dioxide into hydrocarbon fuels. *Energy Environ. Sci.* **3,** 1311-1315 (2010).

11. Wang, V. et al. Vaspkit: A user-friendly interface facilitating high-throughput computing and analysis using VASP code. *Comput. Phys. Commun.* **267,** 108033 (2021).

12. Kim, H. S. et al. Oxygen vacancies enhance pseudocapacitive charge storage properties of MoO_3-x_. *Nat. Mater*. **16,** 454-460 (2017).

13. Lin, L. et al. Reversing sintering effect of Ni particles on γ-Mo_2_N via strong metal support interaction. *Nat. Commun*. **12,** 6978 (2021).

14. Lin, L. et al. Atomically dispersed Ni/α-MoC catalyst for hydrogen production from methanol/water. *J. Am. Chem. Soc.* **143,** 309-317 (2021).

15. Yao, S. et al. Exploring metal–support interactions to immobilize subnanometer Co clusters on γ–Mo_2_N: A highly selective and stable catalyst for CO_2_ activation. *ACS Catal*. **9,** 9087-9097 (2019).

16. Zaman, S. F. et al. Carbon monoxide hydrogenation on potassium promoted Mo_2_N catalysts. *Appl. Catal. A: Gen*. **532,** 133-145 (2017).

17. Binnewies, M. et al. Thermochemical data of elements and compounds. *Weinheim: Wiley-VCH*. ed. 2nd. (2002).

18. Chase, M. W. NIST-JANAF thermochemical tables. *American Institute of Physics* ed. 4. (1998).

19. Han, N. et al. Ultrathin bismuth nanosheets from in situ topotactic transformation for selective electrocatalytic CO_2_ reduction to formate. *Nat. Commun.* **9,** 1320 (2018).
